# Prediction of adolescent weight status by machine learning: a population-based study

**DOI:** 10.1186/s12889-024-18830-1

**Published:** 2024-05-20

**Authors:** Hengyan Liu, Yik-Chung Wu, Pui Hing Chau, Thomas Wai Hung Chung, Daniel Yee Tak Fong

**Affiliations:** 1 School of Nursing, The University of Hong Kong, 3 Sassoon Road, Pokfulam, Hong Kong PR China; 2 Department of Electrical and Electronic Engineering, The University of Hong Kong, Hong Kong, PR China; 3 Family & Student Health Branch, Department of Health, Hong Kong, PR China

**Keywords:** Obesity, Overweight, Adolescent health, Machine learning, Prediction

## Abstract

**Background:**

Adolescent weight problems have become a growing public health concern, making early prediction of non-normal weight status crucial for effective prevention. However, few temporal prediction tools for adolescent four weight status have been developed. This study aimed to predict the short- and long-term weight status of Hong Kong adolescents and assess the importance of predictors.

**Methods:**

A population-based retrospective cohort study of adolescents was conducted using data from a territory-wide voluntary annual health assessment service provided by the Department of Health in Hong Kong. Using diet habits, physical activity, psychological well-being, and demographics, we generated six prediction models for successive weight status (normal, overweight, obese and underweight) using multiclass Decision Tree, Random Forest, k-Nearest Neighbor, eXtreme gradient boosting, support vector machine, logistic regression. Model performance was evaluated by multiple standard classifier metrics and the overall accuracy. Predictors’ importance was assessed using Shapley values.

**Results:**

442,898 Primary 4 (P4, Grade 4 in the US) and 344,186 in Primary 6 (P6, Grade 6 in the US) students, with followed up until their Secondary 6 (Grade 12 in the US) during the academic years 1995/96 to 2014/15 were included. The XG Boosts model consistently outperformed all other model in predicting the long-term weight status at S6 from P4 or P6. It achieved an overall accuracy of 0.72 or 0.74, a micro-averaging AUC of 0.92 or 0.93, and a macro-averaging AUC of 0.83 or 0.86, respectively. XG Boost also demonstrated accurate predictions for each predicted weight status, surpassing the AUC values obtained by other models. Weight, height, sex, age, frequency and hours of aerobic exercise were consistently the most important predictors for both cohorts.

**Conclusions:**

The machine learning approaches accurately predict adolescent weight status in both short- and long-term. The developed multiclass model that utilizing easy-assessed variables enables accurate long-term prediction on weight status, which can be used by adolescents and parents for self-prediction when applied in health care system. The interpretable models may help to provide the early and individualized interventions suggestions for adolescents with weight problems particularly.

**Supplementary Information:**

The online version contains supplementary material available at 10.1186/s12889-024-18830-1.

## Background

Adolescence is defined as a unique decade of human development by the World Health Organization. It is a life stage when growth spurts, puberty changes and the major non-communicable diseases (NCDs) start or are reinforced [1, 2]. However, the Lancet Commission on Adolescent Health and Well-being indicated that global health and social policy have overlooked adolescent health, resulting in fewer health improvements compared to other age groups [3, 4]. Among the various health concerns during adolescence, weight problems are particularly prevalent, with obesity remaining a serious health challenge in many countries.

Overweight and obesity are strongly associated with NCDs and are considered decisive risk factors for premature mortality and physical morbidity in later life. Astonishingly, 80% of obese adolescents remain obese in later adulthood [5, 6]. On the other hand, being underweight in adolescence is associated with psychiatric disorders [7], osteoporosis [8], scoliosis [9], and pubertal delay [10]. In Hong Kong, the prevalence of overweight and obesity among 9-to-12-year-old students increased from 20% in 1999/2000 to 25% in 2008/9 [11]. This prevalence spiked even further to 24.1% during the covid-19 pandemic in 2020, largely attributed to lifestyle changes [12]. Additionally, 20.5% of 12-to-18-year-old students reported being mildly or severely underweight in 2007 [13]. Therefore, controlling weight problems during adolesence is a paramount public health issue.

Studies have suggested that weight problems during adolescence can often be prevented by strategies that are more cost-effective than clinic-based weight-loss programs [14]. Early intervention is crucial to control the adolescent obesity epidemic [15]. Thus, NCD-related health behaviors, such as weight management among adolescents, deserve more attention to prevent future disease development [3]. Predictive models that can accurately classify a child’s future weight status would be valuable tools for tackling child and adolescent weight problems early.

While logistic regression (LG) has traditionally been used to predict adolescent weight status, it is limited to binary outcomes and a specific structural form of the predictors which may result in suboptimal prediction accuracy [16, 17]. In contrast, machine learning (ML) algorithms can accommodate multiclass outcomes and fully consider the complex interrelationships among all predictors by eliciting all possible patterns and thus may optimize the prediction accuracy [18]. As a result, ML models have become increasingly popular. However, the latest review of ML models revealed that many studies only considered the cross-sectional classification rather than temporal prediction [18, 19]. Moreover, most temporal prediction models used birth, infant, or parental measurements to predict overweight or obesity in early childhood period [19, 20]. Only one study derived a deep learning prediction model for adolescents, but it only focused on predicting obesity for three subsequent years [21]. Thus, there has been neither a prediction model that utilizes ML to predict multiclass weight statuses (underweight, normal, overweight, and obese) for more than three years in adolescence, nor a ML-based prediction models of weight status for the Chinese population.

Therefore, we aimed to develop ML models to predct weight status in children, which can assist health professionals in identifying those who are at risk of developing weight problems. We evaluated the performance of these models in a large population-based cohort of children in Hong Kong, and validated them in an independent cohort. We also assessed the relative importance of the predictors to provide more evidence on early weight problems intervention practices.

## Methods

### Design and setting

We conducted a retrospective cohort study of P4 students from the 1995/1996 to 2015/2016 academic cohorts, who were followed until Secondary 6 (S6, Grade 12 in the US). P4 students are cognitively competent to provide self-reported measurements [22]. Additionally, we chose a cohort of P6 students from 1995/1996 to 2013/14 academic cohorts to predict weight status after P6, the last year of primary education in Hong Kong before students are promoted to the secondary level. Students who visited at least two years and had completed health measurements records were included. Data were obtained from the Student Health Service (SHS) of the Department of Health in Hong Kong, which has provided voluntary territory-wide annual health assessment services for primary and secondary students since 1995/1996. The health assessment questionnaire changed in 2015/16 [23]. Therefore, we included P4 students during 1995/1996 to 2014/2015, allowing at least one year of follow-up prediction. Fruther details of the survey health assessment scheme can be found elsewhere [24, 25]. 

### Potential predictor variables

Weight was measured to the nearest 0.1 kg and height to the nearest 0.1 cm were assessed annually at the SHS by well trained healthcare workers or nurses according to the study protocol. Demographics included sex, age and family socioeconomic level. Family’s socioeconomic status was indicated by parental educational level, parental occupation and the type of housing [26]. 

Dietary habits were assessed by “breakfast eating habit,” “sweetness preference during past 7 days,” “junk food intake habit,” “fruit/vegetable intake,” and “milk consumption habit”. Physical activity behaviors were assessed by “frequency of aerobic exercise each week,” “hours of doing aerobic exercise each week,” and “daily hours of TV viewing”. All of these predictors in the structured questionnaires had four response options representing different degrees of frequency or duration. Breakfast habits were assessed by the item ‘I usually have breakfast at?’, we considered three response categories: (i) ‘home’, representing frequently eating at home, (ii) ‘rarely at home’, after combining the original categories of ‘fast food stall/cafeteria/restaurant’ and ‘some other places’, and (iii) ‘no breakfast at all’, representing never eating at home. Thus, this item can be considered an assessment of the frequency of breakfast eating at home.

Psychological development was assessed using the 60-item self-reported Culture Free Self-Esteem Inventory for Children Questionnaire (CFSEI-2), which has been validated in Hong Kong children and adolescents [27, 28]. The Self-Esteem Inventory (SEI) comprises a total score and four domain scores: (i) ‘general self-esteem’ denoting children’s overall perception of themselves, the score ≤ 7 was considered as “very-low”; (ii) ‘social self-esteem’ denoting children’s perception of their peer relationship, (iii) ‘school-related self-esteem’ denoting children’s perception on their ability to achieve academic success, (iv) ‘parent-related self-esteem’ denoting children’s perception on their family’s thoughts. Scores ≤ 2 in any of these three subscales were considered “very-low” [27]. Children with a total score ≤ 19 or a “very-low” score in any domain were considered to have low self-esteem. A lie scale score was also obtained, and a score ≤ 2 indicates the corresponding child’s self-reported assessment is unreliable [27]. 

Potential behavioral problems of children and adolescents were assessed using the 4-item Rutter Behavior Questionnaire (RBQ), which has been validated in Hong Kong children [29]. It inquired about behaviors on hyperactivity, conduct, and emotional disturbances and were completed by parents. A RBQ total score ≥ 19 indicated a potential behavior problem [30]. In total, 25 predictors were considered as input variables in developing multiclass prediction models.

### Prediction outcome

Prediction weight status was classified as normal, obese, overweight, and underweight, based on the next measurement year of the body mass index (BMI, expressed in kg/m^2^) and the age- and sex-specific BMI references in the international Obesity Task Force Standards (IOTF).

### Data preparation

Children with a lie self-esteem score ≤ 2 were considered unreliable and removed. For the type of housing and parental occupation, we ordered their response categories in order of socioeconomic level by using the median monthly domestic household income for each type of housing and occupation obtained from the Hong Kong Census and Statistics Department. Sex as categorical variables was one-hot encoded. The responses of dietary and physical activity behavioral measurements were treated as ordinal variables, and other predictors were considered as continuous variables. Missing data on socioeconomic status were filled out according to the information reported in the student’s other assessment years. The other measurements had less than 5% missing data, which was considered inconsequential to the validity of the model development [31]. We used k nearest neighbour imputation algorithms to the training and test sets separatly to facilitate the use of ML that required complete data [32]. 

### Data analysis

Categorical data were expressed as the number with a percentage for each weight status and compared using chi-square test. Numberical data were presented as the mean ± standard deviation (SD).

### Multiclass prediction models development

P4 students were randomly divided into a training set and a test set at an 80:20 ratio. Multiclass prediction models were developed using the P4 training data to predict weight status in each subsequent year until S6, creating eight prediction windows. We used the same procedure to develop prediction models for the P6 training cohort, creating six prediction windows until S6. The weight status in our cohorts was imbalanced, with underweight, overweight and obese categories being underpresented. The imbalance could have led to biased model performance, where the model may have been more accurate at predicting the majority weight status while performing poorly on the minority weight status. To address this issue, we used the Synthetic Minority Oversampling Technique (SMOTE) sampling technique to the training sets [33]. SMOTE was a widely used technique that creates synthetic samples for the minority categories by generating new instances that are similar to the original underpresented categories. We attempted several ML approaches, including Decision Tree (DT), Random Forest (RF), Supportive Vector Machine (SVM), k-Nearest Neighbor (k-NN), and eXtreme Gradient Boosting (XG Boost), as well as the LG approach for comparison. The short- and long-term prediction abilities of the models were compared by calculating the correct classification rate, overall accuracy of the test set and micro-, macro-averaging area under the curve (AUC). Receiver operating characteristics (ROC) curves for each weight status on test set were also obtained. The AUC, precision, recall and F1-score were calculated to evaluate the model prediction accuracy, and assess the ability to predict an abnormal weight status. The precision and recall are conceptually equivalent to the sensitivity and positive predictive value, and the F1 score is the harmonic mean of precision and recall [34]. For predicting a specific weight status, all accuracy measures ranged from 0 to 1, with a higher value indicating a higher accuracy.

**Fig. 1:**
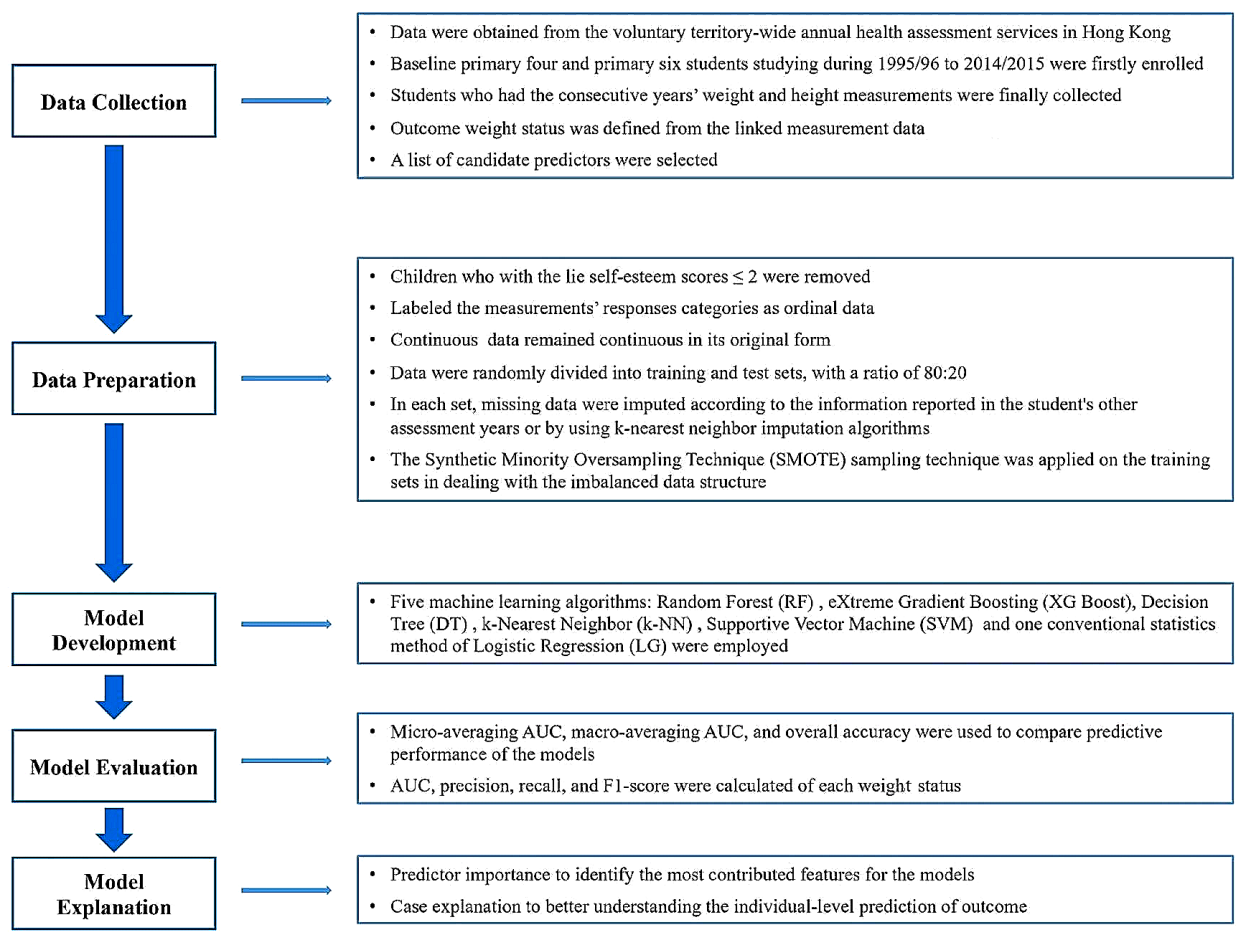
Graphical illustration of the workflow used for this study

To examine the importance of each predictor at both population and individual levels, based on the best performing prediction models, we used the Shapley Additive Explanations (SHAP) to obtain their contributions for a prediction window [35]. SHAP value is assigned to each predictor and can quantify them by comparing the differences with and without that predictor. The Shapley values from all prediction windows in each cohort were used to compare the summary importance of predictors by different weight status. Furthermore, to better understand the individual-level prediction of weight status, we selected two students as examples and used SHAP waterfall plots to illustrate the importance of different predictors for each student. Figure 1 shows the workflow used for this study. All prediction models were developed and compared using Python software (version 3.10) with Scikit-Learn.

## Results

A total of 442,898 and 344,186 students were enrolled in P4 and P6 from 1995/96 to 2014/15. The characteristics of the students in these two cohorts are shown in Table 1. The number of students in successive prediction windows (indicated by academic grade) decreased due to the loss of follow-up. Of the enrolled students in P4 and P6, respectively 224,398 (50.7%) and 171,768 (49.9%) were male. The mean age for the two cohorts were 9.4 ± 0.56 and 11.3 ± 0.54 years, respectively. The prevalence of normal weight, underweight, overweight, and obese children were, respectively 63.4%, 12.0%, 18.5%, and 6.0% at P4, and 65.5%, 11.9%, 18.5%, and 4.1% at P6. The characteristics of demographic, personal lifestyle, and psychological wellbeings among different weight status are also summarized and compared in the Supplementary Tables S1 and S2. All predictors showed significant difference across different weight status in both the P4 and P6 cohorts.

**Table 1:** Baseline characteristics of students in the primary four and primary six cohorts

Characteristics		Primary four enrollment group	Primary six enrollment group
		*N*_1_ = 442 898	*N*_2_ = 344 186
**Academic grade of follow-up measurement**			
	Primary Five	442 898	-
	Primary Six	414 190	-
	Secondary One	362 923	344 186
	Secondary Two	286 049	275 428
	Secondary Three	213 818	214 404
	Secondary Four	162 306	172 890
	Secondary Five	107 754	115 418
	Secondary Six	60 274	62 503
**Sex**			
	Male	224 398 (50.7)	171 768 (49.9)
	Female	218 500 (49.3)	172 418 (50.1)
**Age**, *mean (SD), years*		9.4 (0.56)	11.3 (0.54)
**Weight**, *mean (SD), kg*		33.6 (8.03)	42.3 (9.95)
**Height**, *mean (SD), cm*		137.5 (6.80)	149.6 (7.55)
**Weight Status**			
	Normal	280 998 (63.4)	225 294 (65.5)
	Underweight	53 100 (12.0)	40 950 (11.9)
	Overweight	82 154 (18.5)	63 697 (18.5)
	Obese	26 646 (6.0)	14 245 (4.1)
**Breakfast Eating Habit**			
	Missing value	430 (0.1)	294 (0.1)
	home	385 741 (87.1)	290 519 (84.4)
	rarely at home	35 321 (8.0)	31 098 (9.0)
	no breakfast	21 406 (4.8)	22 275 (6.5)
**Sweetness Preference during Past 7 days**			
	Missing value	1 098 (0.2)	606 (0.2)
	0–3 times	145 145 (32.8)	103 828 (30.2)
	4–6 times	252 972 (57.1)	216 177 (62.8)
	once daily	29 545 (6.7)	16 584 (4.8)
	2 times or above daily	14 134 (3.2)	6 991 (2.0)
**Junk Food Intake Habits**			
	Missing value	1 128 (0.3)	683 (0.2)
	every day	23 345 (5.3)	19 490 (5.7)
	Occasionally	267 862 (60.5)	220 262 (64.0)
	Rarely	143 752 (32.5)	100 013 (29.1)
	Never	6 797 (1.5)	3 738 (1.1)
**Fruit/ vegetable Intake**			
	Missing value	18 190 (4.1)	16 373 (4.8)
	at least thrice a day	111 640 (25.2)	74 090 (21.5)
	once or twice a day	239 839 (54.2)	196 348 (57.0)
	once every few days	57 000 (12.9)	48 311 (14.0)
	less than once a week	16 229 (3.7)	9 064 (2.6)
**Milk Consumption Habit**			
	Missing value	1 459 (0.3)	795 (0.2)
	at least once a day	39 480 (31.5)	89 464 (26.0)
	once every few days	130 011 (29.4)	103 221 (30.0)
	less than once a week	85 475 (19.3)	79 272 (23.0)
	Never	87 283 (19.7)	71 614 (20.8)
**Frequency of Aerobic Exercise**			
	Missing value	7 096 (1.6)	4 692 (1.4)
	at least thrice a day	133 299 (30.1)	95 665 (27.8)
	once or twice a day	199 672 (45.1)	162 209 (47.1)
	once every few days	73 430 (16.6)	48 311 (14.0)
	less than once a week	29 446 (6.6)	19 736 (5.7)
**Hours of Aerobic Exercise**			
	Missing value	7 218 (1.6)	5 047 (1.5)
	more than an hour	142 732 (32.2)	132 588 (38.5)
	half to one hour	175 401 (39.6)	125 159 (36.4)
	less than half an hour	83 715 (18.9)	58 791 (17.1)
	zero	33 832 (7.6)	22 601 (6.6)
**Daily Hours of TV Viewing**			
	Missing value	5 198 (1.2)	3 808 (1.1)
	less than an hour	89 950 (20.3)	49 477 (14.4)
	one to two hours	175 870 (39.7)	131 494 (38.2)
	two to four hours	119 688 (27.0)	114 849 (33.4)
	more than four hours	52 101 (11.8)	44 558 (12.9)
**SEI Score**^**a**^, *mean (SD)*			
	Total	36.96 (6.94)	37.73 (6.98)
	General	14.85 (3.26)	16.28 (3.24)
	Social	6.49 (1.82)	6.89 (1.79)
	School-related	7.06 (1.84)	7.02 (1.91)
	Lie (exclude score ≤ 2)	5.92 (1.71)	6.18 (1.61)
	Parent-related	8.56 (1.87)	8.54 (1.95)
**RBQ Score**^**b**^, *mean (SD)*			
	Total	9.38 (5.84)	8.47 (5.49)
	Conduct	1.52 (1.31)	1.42 (1.26)
	Emotion	1.40 (1.26)	1.39 (1.24)
	Hyperactivity	1.76 (1.56)	1.38 (1.45)
**Educational Level of Student’s Father**			
	Missing value	19 045 (4.3)	13 767 (4.0)
	No Schooling	2 657 (0.6)	2 065 (0.6)
	Kindergarten	443 (0.1)	344 (0.1)
	Primary	64 220 (14.5)	52 316 (15.2)
	Lower Secondary	93 894 (21.2)	73 656 (21.4)
	Upper Secondary	177 159 (40.0)	139 051 (40.4)
	Matriculation	17 273 (3.9)	13 423 (3.9)
	Tertiary (Non-degree Course)	17 273 (3.9)	13 767 (4.0)
	Tertiary (Degree Course)	51 376 (11.6)	36 140 (10.5)
**Educational Level of Student’s Mother**			
	Missing value	9 300 (2.1)	6 540 (1.9)
	No Schooling	3 543 (0.8)	3 098 (0.9)
	Kindergarten	443 (0.1)	344 (0.1)
	Primary	64 220 (14.5)	54 037 (15.7)
	Lower Secondary	95 666 (21.6)	72 967 (21.2)
	Upper Secondary	205 505 (46.4)	162 456 (47.2)
	Matriculation	17 273 (3.9)	13 079 (3.8)
	Tertiary (Non-degree Course)	15 059 (3.4)	11 014 (3.2)
	Tertiary (Degree Course)	32 774 (7.4)	21 340 (6.2)
**Occupation of Student’s Father**			
	Missing value	18 602 (4.2)	13 423 (3.9)
	Managers and Administrators	46 947 (10.6)	37 860 (11.0)
	Professionals	22 145 (5.0)	16 521 (4.8)
	Associate Professionals	26 574 (6.0)	21 340 (6.2)
	Clerks	41 190 (9.3)	33 042 (9.6)
	Service Workers and Shop Sales Workers	84 594 (19.1)	64 019 (18.6)
	Craft and Related Workers	66 435 (15.0)	54 726 (15.9)
	Plant & Machine Operators and Assemblers	66 878 (15.1)	53 005 (15.4)
	Elementary Occupations	54 034 (12.2)	39 926 (11.6)
	Unemployed	15 944 (3.6)	11 358 (3.3)
**Occupation of Student’s Mother**			
	Missing value	6 643 (1.5)	4 474 (1.3)
	Managers and Administrators	13 287 (3.0)	9 637 (2.8)
	Professionals	7 529 (1.7)	5 507 (1.6)
	Associate Professionals	19 045 (4.3)	14 800 (4.3)
	Clerks	73 964 (16.7)	57 479 (16.7)
	Service Workers and Shop Sales Workers	55 805 (12.6)	38 549 (11.2)
	Craft and Related Workers	4 872 (1.1)	3 786 (1.1)
	Plant & Machine Operators and Assemblers	1 772 (0.4)	1 377 (0.4)
	Elementary Occupations	19 045 (4.3)	13 767 (4.0)
	Unemployed	241 379 (54.5)	191 712 (55.7)
**Type of Housing**			
	Missing value	7 086 (1.6)	5 507 (1.6)
	Block-Self-contained	179 817 (40.6)	141 805 (41.2)
	Block-non-Self-contained	10 630 (2.4)	6 884 (2.0)
	Housing Authority Home Ownership Estate	69 978 (15.8)	58 167 (16.9)
	Housing Authority/Society Blocks	154 571 (34.9)	116 335 (33.8)
	Village Houses	16 830 (3.8)	12 391 (3.6)
	Institution	4 429 (1.0)	3 442 (1.0)

Data are n (%) unless otherwise stated

^a^. SEI: Culture Free Self-Esteem Inventory for Children Questionnaire

^b^. RBQ: Rutter Behaviour Questionnaire

**Fig. 2:**
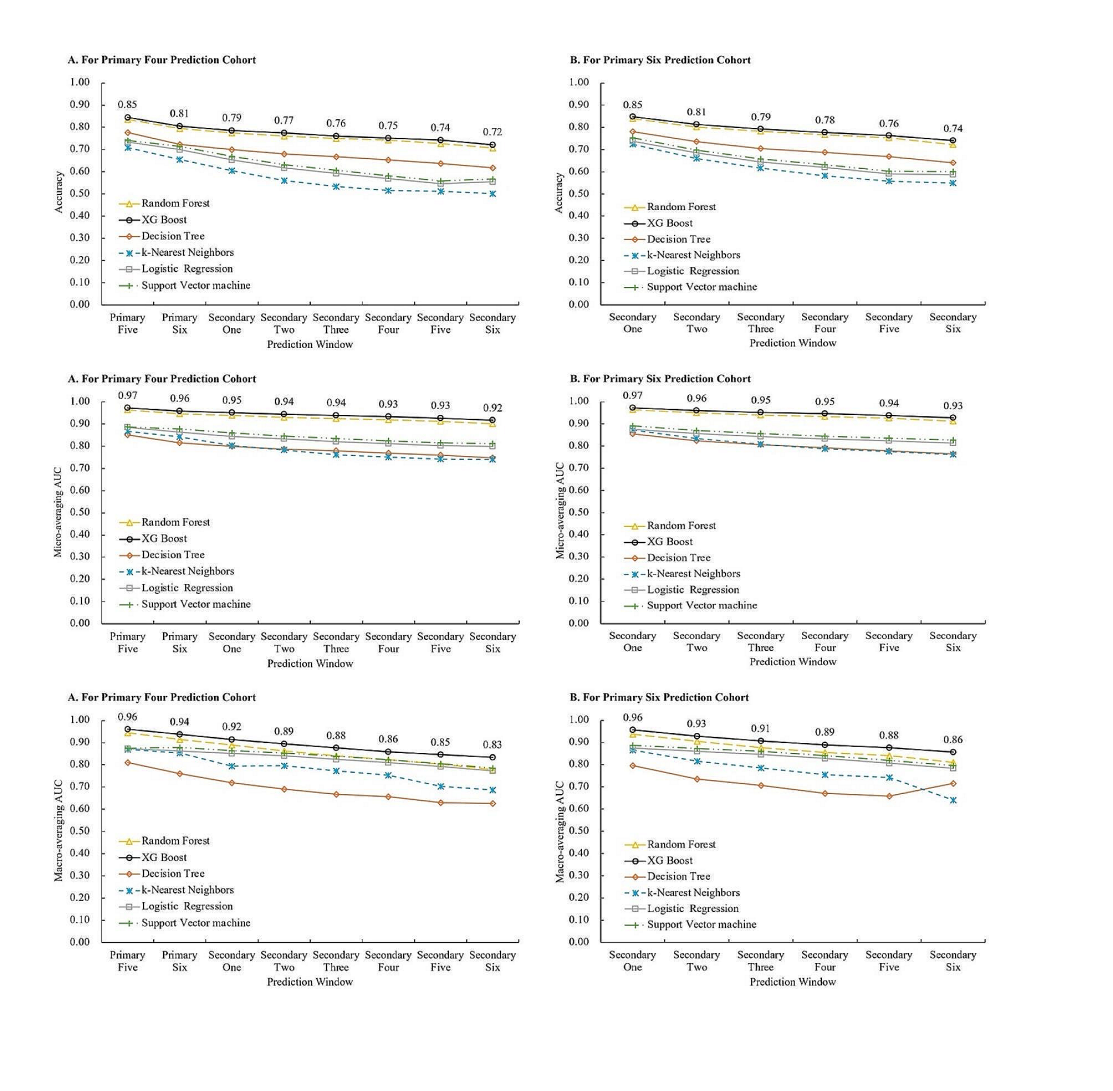
Prediction accuracy of different multiclass machine learning models for every prediction window **A** based on the primary four cohort; **B** based on the primary six cohort; *XG Boost* eXtreme Gradient Boosting

Figure 2 shows the overall predictive ability of the generated prediction models on the test set, with the exact accuracy levels tabulated in Supplementary Table S3. The XG Boost prediction models exhibited higher accuracy than all other models. They demonstrated robust performance in predicting both short- and long-term weight status, with the overall accuracy, micro-averaging AUC, and macro-averaging AUC values exceeding 0.72, 0.92, and 0.83, respectively, for the eight consecutive years of P4 prediction. Similarly, for the six consecutive years of P6 prediction, the corresponding values were greater than 0.74, 0.93, and 0.86, respectively. Table 2 presents the AUC values for each weight status across different models, highlighting XGBoost’s superior performance on multiclass prediction, with AUCs exceeding 0.85 for the underweight group, 0.85 for the overweight group, and 0.92 for the obese group both P4 and P6 predictions. Supplementary Table S4 provides precision, recall and F1-score metrics or each weight status.

**Table 2:** AUC values of different multiclass machine learning models by weight status

(a) based on the primary four cohort									
Weight status	Machine learning model	Primary five	Primary six	Secondary one	Secondary two	Secondary three	Secondary four	Secondary five	Secondary six
Normal	Random forest	0.92	0.88	0.84	0.80	0.76	0.73	0.71	0.69
	XG Boost	0.94	0.90	0.86	0.82	0.79	0.76	0.73	0.72
	Decision Tree	0.80	0.74	0.70	0.67	0.64	0.62	0.60	0.60
	k-Nearest Neighbors	0.84	0.80	0.75	0.73	0.69	0.67	0.63	0.62
	Logistic Regression	0.69	0.68	0.67	0.65	0.61	0.58	0.51	0.47
	Support Vector machine	0.73	0.72	0.71	0.70	0.66	0.63	0.56	0.52
Underweight	Random Forest	0.95	0.93	0.90	0.87	0.86	0.84	0.84	0.83
	XG Boost	0.97	0.95	0.93	0.91	0.89	0.87	0.86	0.85
	Decision Tree	0.81	0.77	0.72	0.69	0.68	0.68	0.67	0.67
	k-Nearest Neighbors	0.90	0.88	0.84	0.84	0.81	0.80	0.76	0.76
	Logistic Regression	0.96	0.94	0.93	0.91	0.89	0.87	0.86	0.85
	Support Vector machine	0.91	0.89	0.88	0.86	0.84	0.82	0.81	0.80
Overweight	Random Forest	0.94	0.90	0.88	0.85	0.84	0.82	0.81	0.80
	XG Boost	0.95	0.92	0.90	0.88	0.87	0.86	0.86	0.85
	Decision Tree	0.79	0.73	0.69	0.66	0.64	0.63	0.62	0.62
	k-Nearest Neighbors	0.86	0.83	0.77	0.76	0.75	0.74	0.71	0.69
	Logistic Regression	0.86	0.86	0.84	0.84	0.84	0.84	0.85	0.83
	Support Vector machine	0.82	0.81	0.81	0.80	0.80	0.80	0.79	0.79
Obese	Random Forest	0.97	0.96	0.94	0.93	0.91	0.90	0.86	0.80
	XG Boost	0.99	0.98	0.97	0.97	0.96	0.95	0.94	0.92
	Decision Tree	0.85	0.80	0.76	0.75	0.70	0.70	0.63	0.62
	k-Nearest Neighbors	0.89	0.91	0.82	0.86	0.84	0.81	0.72	0.68
	Logistic Regression	0.98	0.98	0.97	0.96	0.96	0.95	0.94	0.94
	Support Vector machine	0.94	0.93	0.93	0.92	0.92	0.91	0.89	0.89
**(b) based on the primary six cohort**									
Normal	Random Forest	0.91	0.86	0.81	0.78	0.75	0.72		
	XG Boost	0.93	0.88	0.84	0.81	0.78	0.75		
	Decision Tree	0.79	0.73	0.68	0.66	0.63	0.62		
	k-Nearest Neighbors	0.84	0.78	0.74	0.71	0.68	0.66		
	Logistic Regression	0.68	0.66	0.62	0.57	0.51	0.47		
	Support Vector machine	0.73	0.70	0.67	0.62	0.56	0.52		
Underweight	Random Forest	0.94	0.90	0.88	0.86	0.85	0.83		
	XG Boost	0.95	0.92	0.90	0.89	0.88	0.87		
	Decision Tree	0.78	0.71	0.68	0.65	0.64	0.63		
	k-Nearest Neighbors	0.85	0.80	0.77	0.75	0.74	0.73		
	Logistic Regression	0.88	0.88	0.88	0.88	0.87	0.84		
	Support Vector machine	0.83	0.83	0.83	0.83	0.82	0.79		
Overweight	Random Forest	0.94	0.91	0.89	0.87	0.86	0.84		
	XG Boost	0.96	0.94	0.92	0.90	0.88	0.86		
	Decision Tree	0.80	0.74	0.73	0.70	0.69	0.69		
	k-Nearest Neighbors	0.90	0.86	0.83	0.81	0.80	0.78		
	Logistic Regression	0.95	0.93	0.91	0.90	0.88	0.87		
	Support Vector machine	0.91	0.89	0.87	0.86	0.84	0.83		
Obese	Random Forest	0.96	0.95	0.93	0.91	0.90	0.84		
	XG Boost	0.99	0.98	0.97	0.97	0.96	0.94		
	Decision Tree	0.83	0.76	0.73	0.68	0.67	0.63		
	k-Nearest Neighbors	0.87	0.82	0.79	0.76	0.76	0.70		
	Logistic Regression	0.99	0.98	0.97	0.97	0.96	0.96		
	Support Vector machine	0.94	0.94	0.93	0.93	0.91	0.91		

*AUC* area under the curve; *XG Boost* eXtreme Gradient Boosting

**Fig. 3:**
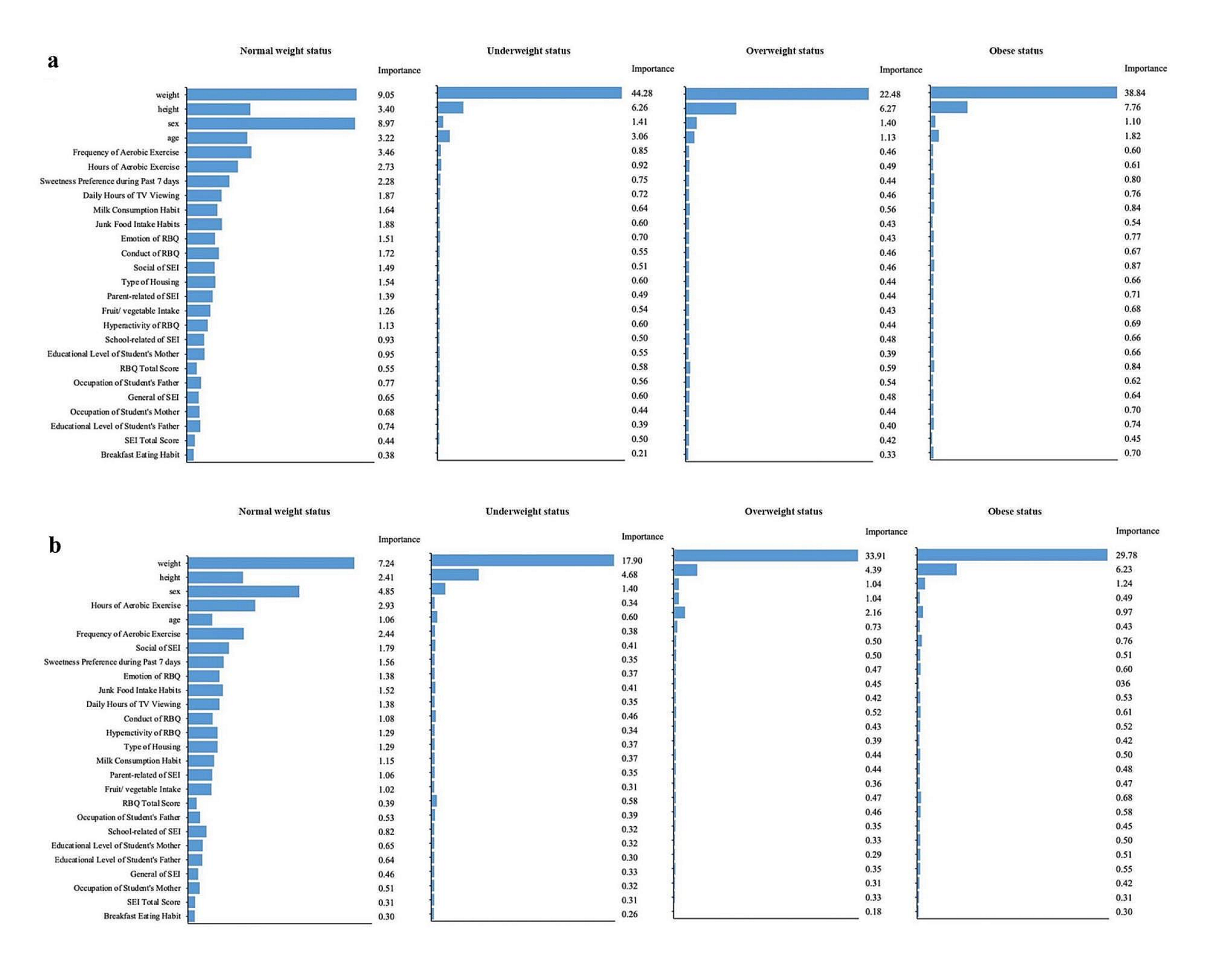
Relative importance of predictors by predicted weight status **(a)** based on the primary four cohort; **(b)** based on the primary six cohort. The relative predictor importance on each weight status was measured by the Shapley values under a XG Boost model. The predictors were ordered in descending order of overall importance

The predictor importance of all 26 variables were evaluated using Shapley values for different weight statuses (Fig. 3).The summary predictor importance by SHAP was presented in a column list in descending order for each cohort. Weight, height, sex, age, frequency and hours of aerobic exercise consistently showed higher importance in the XG Boost prediction models. To further explore the predictive power of these top predictors, we re-developed the XG Boost prediction models using the above six and top three predictors for the P4 and P6 cohorts, respectively, in the training set. However, the models showed reduced accuracy when tested on the test set (Supplementary Table S5).

**Fig. 4:**
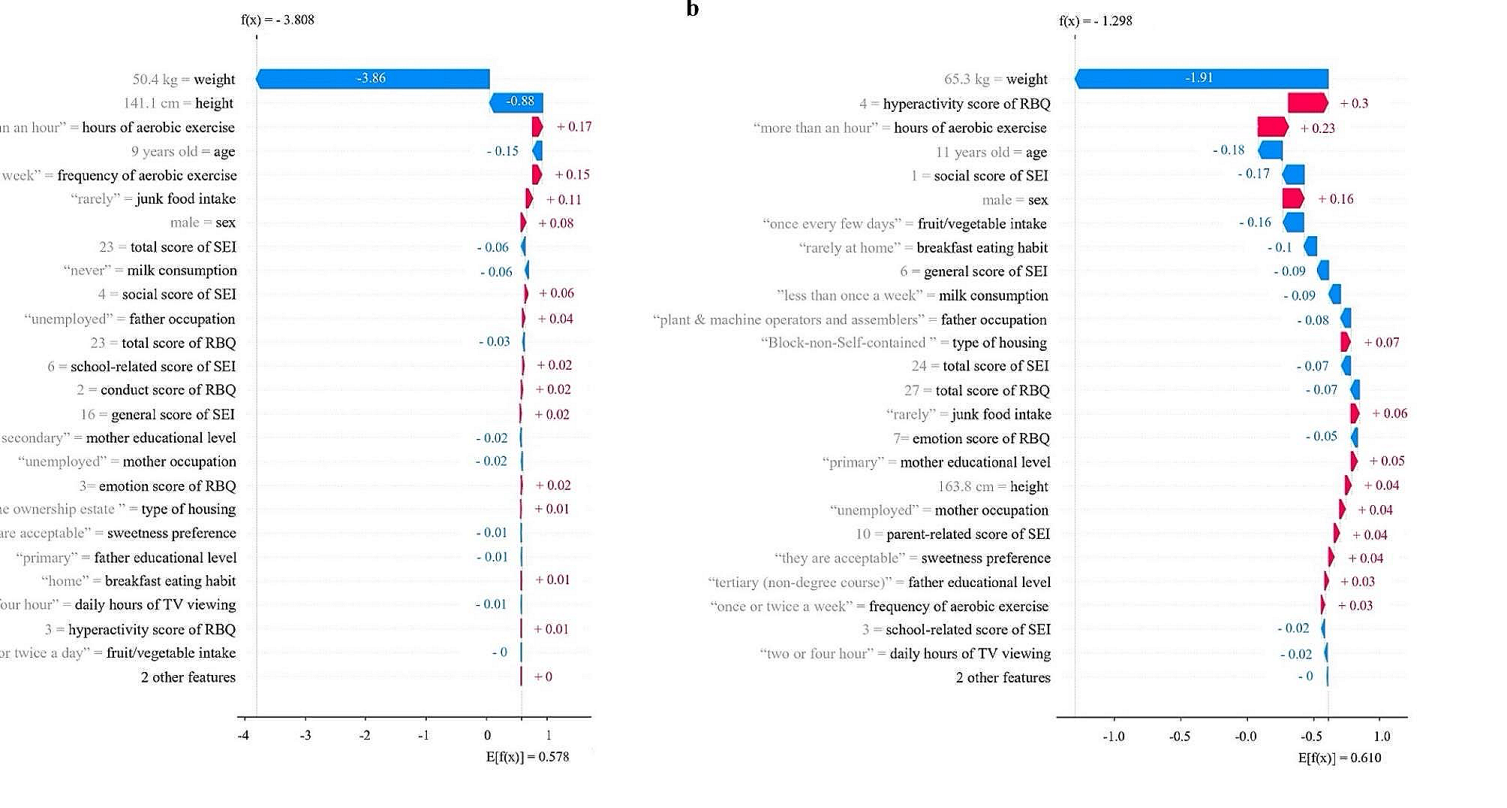
SHAP Waterfall plots of the contribution of each predictor to a predicted weight status **a** Based on a child at primary four data, who is predicted to be obese at secondary one; **b** Based on a child at primary six, who is predicted to be obese at secondary six; Each arrow shows the magnitude and direction a predictor’s contribution to the predicted outcome

To provide a more detailed understanding of the predictors’ contributions to the predictions, we generated Waterfall plots for two children, one at P4 and the other at P6, who were both predicted to be obese at S1 and S6, respectively (Fig. 4). Each arrow in the plot represented the extent and direction of predictor’s contribution to the prediction. An arrow pointing to the left indicated that the corresponding predictor would increase the risk of obesity, whereas an arrow pointing to the right indicated that the predictor would reduce the risk of obesity. The student in Fig. 4a had weight as the main contributor to the predicted outcome of being obese, and weight reduction would be the target for reducing the risk of obesity at S1. In contrast, the student in Fig. 4b also had hyperactivity and hours of aerobic exercises as the main contributors to the prediction, and alleviating hyperactivity and increases hours of aerobic exericses would also be the targets for reducing obesity at S6.

## Discussion

To the best of our knowledge, this is the first study to simultaneously predict four weight statuses (normal weight, underweight, overweight, and obese) using ML, for both short- and long-term prediction. Our large population-based cohort of children around 9 or 11 years old, followed until around 17 years of age, allowed us to develop and validate these models with high accuracy. The use of ML in predicting weight status demonstrated superior accuracy compared to traditional methods, providing a preview of the weight status over subsequent years. Our models offer potential benefits for health professionals in identifying children who are at risk of developing weight problems.

In our study, the XG Boost machine-learning method demonstrated the highest accuracy for predicting weight status in adolescents for all prediction windows. Our models using P4 data to predict weight status at P5 to S6 reached a micro-averaging AUC of 0.97 to 0.92, while using P6 data for prediction until S6 had a micro-averaging AUC of 0.97 to 0.93. These accuracies were higher than those achieved by LG (0.88 to 0.80 and 0.88 to 0.81, respectively). The suboptimal accuracy of LG was also shown in a previous study which predicted overweight at 2 years [36]. Our large population-based sample made multiclass prediction possible by ensuring a decent number of children at each weight status. To our knowledge, no prediction models have been developed for the simultaneous temporal prediction of multiclass weight status in adolescents. Our XG Boost models had better performance for at least six prediction years, indicating their long-term prediction ability. However, the predictive ability was gradually declined as the time span extend, which can be attributed to the diminishing influence of the predictors over a longer period. Moreover, the corresponding AUCs for each abnormal weight status were consistently above 0.85 at every prediction year. Therefore, our prediction models using XG Boost can accurately predict all weight statuses for adolescents at around 9 and 11 year-old for the subsequent years during adolescence.

We evaluated several ML algorithms for predicting weight status in adolescents and found that the SVM approach performed slightly better than LG, while it was not appropriate due to extremely long computation time. The RF generally outperformed LG, while k-NN and DT showed unstable prediction abilities and yielded less consistent results. Each ML algorithm may have its various advantages and disadvantages, and the performance may vary depending on the application. Thus, we attempted several ML algorithms on a large population-based sample to allow the robust assessment of various prediction approaches. In our study, XG Boost was the most effective tool for predicting weight status in adolescents due to its ability to handle nonlinear predictors, and its high computing efficiency using parallel computing.

To gain a deeper understanding of the predictors influcening adolescent weight status, we repeated the same model development process on two cohorts. Although there is apparent overlap between the important predictors across the two cohorts, there are also some distinct differences. Notably, three subscares of the RBQ, particularly emotion and hyperactivity, had increased contributions to prediction in the P6 cohort compared to P4. Additionaly, the social subscale of the SEI had increased importance in the P6 cohort. The P6 cohort was designed for the prediction of weight status in secondary school students who are at least two years older than the children in the P4 cohort. One possible reason for these differences is that the transition from primary to secondary school may be a particularly difficult experience for some children [37]. The adjustment to a new social environment can lead to anxiety and emotional problems, which can lower their social self-esttem if they fail to negotiate new relationships.

Our findings also suggest that emotoional and behavioral problems, as well as low self-esteeem, are associated with weight problems in adolescents. Adolescents with emotional and behavioral problems are more susceptible to losing behavioral control, disordered eating, and sedentary behaviors, leading to poor weight management [38]. In addition, individuals with lower self-esteem tend to experience painful self-awareness, do less future planning, have increased food consumption, and decreased physical activity, leading to a higher risk of being overweight or obese [39, 40]. These findings highlight the need for early intervention in adolescents with emotional and behavorial problems and low self-esteem to prevent or manage weight problems.

Historical weight and height are the most crucial predictors of weight status during most of adolescence. Previous ML prediction models considered these measurements only at birth or predicted weight status in at most three subsequent years [19]. Age and sex are also significant predictors commonly used to predict adolescent weight status, and our study found that the averaged Shaply values of weight, height and sex were consistently quite high. However, the accuracy of the models decreased when we excluded other predictors, indicating that all selected predictors should be considered for optimal early intervention for weight problems in Hong Kong adolescents, especially physical activity habits.

ML models may also offer a powerful tool for prioritizing predictors that are most relevant to predicting outcomes for adolescents. In our study, each student may have a unique set of predictors that contribute to the predicted outcome. To determine the importance of each predictor, we use the Shapley value, which is represented in the Waterfall plot as the contribution of each predictor to the final predicted outcome. For instance, consider our illustrative example with two students, one from primary four and the other from primary six, who have the same predicted higher risk of being obese in a subsequent year. For the P6 students, weight control, addressing hyperactivity, and increasing hours of aerobic exercises are the most critical strategies, while for the P4 student, weight control is the key predictor. By identifying the most influential predictors, our multiclass prediction models can assist health professionals in developing targeted and effective interventions to prevent obesity in adolescents.

### Limitations

Our study has some limitations. First, we did not consider all the relevant predictors, such as parental weight status and lifestyle, which have been shown to influence adolescents’ BMI [41]. Future studies could include more predictors to improve model accuracy. Second, our retrospective design limited data quality control. However, the annual health assessment scheme data were well-managed by the Department of Health, allowing us to apply ML for multiclass prediction. Although a prospective design would have been ideal, it was not feasibile to accrue a large cohort with a long follow-up period for applying ML algorithms. Future studies may consider using a prospective design to validate our findings. Third, we did not conduct feature selection to determine the optimal set of predictors for our prediction models. However, all the predictors included in our prediction models showed a significant association with weight status. Their inclusion would enhance the prediction accuracy, particularly for long-term prediction. Nonetheless, feature selection that takes into account the importance, stability across samples, or other performance criteria of the predictors would be desirable in future studies.

## Conclusions

Our study demonstrates the potential of ML approaches for multiclass prediction of child and adolescent weight status in Hong Kong. XG Boost performed better than the other approaches, indicating its potential to improve the accuracy of existing weight status prediction models. Our results suggest that it is possible to predict the long-term weight status by utilizing student characteristics as early as primary four. With the interpretability and high accuracy of the XG Boost models developed in this study, health professionals can improve weight promotion programs and provide personalized and effective weight management interventions for adolescents.

### Electronic supplementary material

Below is the link to the electronic supplementary material.

Supplementary Material 1

## Data Availability

The data supporting the conclusions of this study are available from the Student Health Services, Department of Health, Hong Kong SAR, but restrictions apply to the availability of these data, which were used under agreement for the current study, and so are not publicly available. Data are however available from the authors upon reasonable request and with permission of the Student Health Services, Department of Health, Hong Kong SAR.

## Abbreviations

BMI: Body mass index
SEI: The Self-Esteem Inventory Questionnaire
RBQ: Rutter Behavior Questionnaire
ML: Machine learning
LG: Logistic regression
DT: Decision Tree
RF: Random forest
k-NN: k-nearest neighbor
XG Boost: eXtreme gradient boosting
SVM: Support vector machine
ROC: Receiver operating characteristics
AUC: Area under the receiver operating characteristic curve
SMOTE: Synthetic minority oversampling technique
SHAP: Shapley Additive Explanations
