# Supplementary material for: Prediction of adolescent weight status by machine learning: a population-based study
Source: BMC Public Health. 2024 May 20;24:1351. doi: 10.1186/s12889-024-18830-1 (PMC11103824; doi:10.1186/s12889-024-18830-1)
Supplement: Supplementary file 1 — Supplementary Material 1 [file 12889_2024_18830_MOESM1_ESM.docx]

**Supple. Table S1** Baseline demographics of children by weight status

|  | Primary Four Cohort (N_1_ = 442,898) | | | | | Primary Six Cohort (N_2_ = 344,186) | | | | | |
| --- | --- | --- | --- | --- | --- | --- | --- | --- | --- | --- | --- |
| Variables | Normal Weight (*n* = 280,998) | Underweight (*n* = 53,100) | Overweight (*n* = 82,154) | Obese (*n* = 26,646) | *p* Value | Normal Weight (*n* = 225,294) | Underweight (*n* = 40,950) | Overweight (*n* = 63,697) | Obese (*n* = 14,245) | | *p* Value |
| Male, n (%) | 135,421 (48.2) | 21,719 (40.9) | 48,395 (58.9) | 18,863 (70.8) | < 0.001^‡^ | 105,876 (47.0) | 17,709 (43.0) | 38,128 (59.9) | 10,055 (70.6) | | < 0.001^‡^ |
| Female, n (%) | 145,577 (51.8) | 31,381 (59.1) | 33,759 (41.1) | 7,783 (29.2) | < 0.001^‡^ | 119,418 (53.0) | 23,241 (57.0) | 25,569 (40.1) | 4,190 (29.4) | | < 0.001^‡^ |
| Age, years, mean (SD) | 9.37 (0.55) | 9.45 (0.57) | 9.36 (0.54) | 9.29 (0.52) | < 0.001^‡^ | 11.35 (0.54) | 11.40 (0.56) | 11.29 (0.51) | 11.25 (0.50) | | < 0.001^‡^ |
| Weight, kg, mean (SD) | 31.12 (4.46) | 25.00 (2.64) | 41.32 (4.93) | 50.99 (6.89) | < 0.001^‡^ | 39.78 (5.89) | 30.59 (3.63) | 52.48 (6.21) | 65.16 (8.41) | | < 0.001^‡^ |
| Height, cm, mean (SD) | 136.75 (6.55) | 134.51 (6.19) | 140.10 (6.40) | 142.23 (6.42) | < 0.001^‡^ | 149.43 (7.40) | 145.44 (7.16) | 151.75 (7.04) | 153.71 (7.18) | | < 0.001^‡^ |
| Educational Level of Student's Father, n (%) |  |  |  |  |  |  |  |  |  | |  |
| Missing value | 11,630 (4.1) | 2,085 (3.9) | 3,468 (4.2) | 1,295 (4.9) |  | 8,618 (3.8) | 1,447 (3.5) | 2,575 (4.0) | 681 (4.8) | |  |
| No Schooling | 1,489 (0.5) | 297 (0.6) | 408 (0.5) | 168 (0.6) | < 0.001^‡^ | 1,085 (0.5) | 250 (0.6) | 312 (0.5) | 84 (0.6) | | < 0.001^‡^ |
| Kindergarten | 286 (0.1) | 52 (0.1) | 82 (0.1) | 30 (0.1) | < 0.001^‡^ | 204 (0.1) | 36 (0.1) | 59 (0.1) | 14 (0.1) | | < 0.001^‡^ |
| Primary | 39,717 (14.1) | 7,626 (14.4) | 11,372 (13.8) | 4,163 (15.6) | < 0.001^‡^ | 32,740 (14.5) | 6,245 (15.3) | 9,335 (14.7) | 2,307 (16.2) | | < 0.001^‡^ |
| Lower Secondary | 57,454 (20.4) | 10,672 (20.1) | 17,578 (21.4) | 6,043 (22.7) | < 0.001^‡^ | 46,363 (20.6) | 8,473 (20.7) | 13,497 (21.2) | 3,268 (22.9) | | < 0.001^‡^ |
| Upper Secondary | 110,253 (39.2) | 20,917 (39.4) | 33,409 (40.7) | 10,833 (40.7) | < 0.001^‡^ | 89,902 (39.9) | 16,197 (39.6) | 26,146 (41.0) | 5,846 (41.0) | | < 0.001^‡^ |
| Matriculation | 11,309 (4.0) | 2,199 (4.1) | 3,238 (3.9) | 937 (3.5) | < 0.001^‡^ | 9,360 (4.2) | 1,710 (4.2) | 2,547 (4.0) | 492 (3.5) | | < 0.001^‡^ |
| Tertiary (Non-  degree Course) | 11,830 (4.2) | 2,321 (4.4) | 3,193 (3.9) | 851 (3.2) | < 0.001^‡^ | 9,896 (4.4) | 1,812 (4.4) | 2,580 (4.1) | 440 (3.1) | | < 0.001^‡^ |
| Tertiary (Degree  Course) | 37,030 (13.2) | 6,931 (13.1) | 9,406 (11.4) | 2,326 (8.7) | < 0.001^‡^ | 27,126 (12.0) | 4,780 (11.7) | 6,646 (10.4) | 1,113 (7.8) | | < 0.001^‡^ |
| Educational Level of Student's Mother, n (%) |  |  |  |  |  |  |  |  |  | |  |
| Missing value | 5,380 (1.9) | 937 (1.8) | 1,670 (2.0) | 666 (2.5) |  | 3,704 (1.6) | 681 (1.7) | 1,219 (1.9) | 332 (2.3) | |  |
| No Schooling | 2,225 (0.8) | 462 (0.9) | 627 (0.8) | 218 (0.8) | < 0.001^‡^ | 1,761 (0.8) | 377 (0.9) | 512 (0.8) | 123 (0.9) | | < 0.001^‡^ |
| Kindergarten | 288 (0.1) | 50 (0.1) | 70 (0.1) | 38 (0.1) | < 0.001^‡^ | 251 (0.1) | 38 (0.1) | 61 (0.1) | 15 (0.1) | | < 0.001^‡^ |
| Primary | 39,758 (14.1) | 7,961 (15.0) | 11,403 (13.9) | 3,970 (14.9) | < 0.001^‡^ | 34,304 (15.2) | 6,816 (16.6) | 9,565 (15.0) | 2,249 (15.8) | | < 0.001^‡^ |
| Lower Secondary | 58,936 (21.0) | 10,807 (20.4) | 17,928 (21.8) | 6,130 (23.0) | < 0.001^‡^ | 46,711 (20.7) | 8,341 (20.4) | 13,391 (21.0) | 3,210 (22.5) | | < 0.001^‡^ |
| Upper Secondary | 129,205 (46.0) | 24,627 (46.4) | 38,367 (46.7) | 12,377 (46.4) | < 0.001^‡^ | 105,900 (47.0) | 19,033 (46.5) | 30,305 (47.6) | 6,806 (47.8) | | < 0.001^‡^ |
| Matriculation | 11,517 (4.1) | 2,066 (3.9) | 3,336 (4.1) | 960 (3.6) | < 0.001^‡^ | 9,022 (4.0) | 1,587 (3.9) | 2,558 (4.0) | 491 (3.4) | | < 0.001^‡^ |
| Tertiary (Non-  degree Course) | 10,218 (3.6) | 1,908 (3.6) | 2,761 (3.4) | 743 (2.8) | < 0.001^‡^ | 7,950 (3.5) | 1,380 (3.4) | 2,101 (3.3) | 342 (2.4) | | < 0.001^‡^ |
| Tertiary (Degree  Course) | 23,471 (8.4) | 4,282 (8.1) | 5,990 (7.3) | 1,544 (5.8) | < 0.001^‡^ | 15,691 (7.0) | 2,697 (6.6) | 3,985 (6.3) | 677 (4.8) | | < 0.001^‡^ |
| Occupation of Student's Father, n (%) |  |  |  |  |  |  |  |  |  | |  |
| Missing value | 11,739 (4.2) | 2,140 (4.0) | 3,640 (4.2) | 1,185 (4.4) |  | 8,465 (3.8) | 1,460 (3.6) | 2,484 (3.9) | 621 (4.4) | |  |
| Managers and  Administrators | 30,266 (10.8) | 5,265 (9.9) | 9,327 (11.4) | 2,758 (10.4) | < 0.001^‡^ | 25,307 (11.2) | 4,249 (10.4) | 7,487 (11.8) | 1,530 (10.7) | | < 0.001^‡^ |
| Professionals | 16,124 (5.7) | 3,143 (5.9) | 3,826 (4.7) | 972 (3.6) | < 0.001^‡^ | 12,653 (5.6) | 2,265 (5.5) | 2,865 (4.5) | 486 (3.4) | | < 0.001^‡^ |
| Associate  Professionals | 18,512 (6.6) | 3,625 (6.8) | 4,761 (5.8) | 1,263 (4.7) | < 0.001^‡^ | 15,273 (6.8) | 2,798 (6.8) | 3,856 (6.1) | 692 (4.9) | | < 0.001^‡^ |
| Clerks | 26,487 (9.4) | 5,348 (10.1) | 7,445 (9.1) | 2,283 (8.6) | < 0.001^‡^ | 21,824 (9.7) | 4,170 (10.2) | 5,930 (9.3) | 1,278 (9.0) | | < 0.001^‡^ |
| Service Workers  and Shop Sales  Workers | 52,208 (18.6) | 9,858 (18.6) | 15,778 (19.2) | 5,321 (20.0) | < 0.001^‡^ | 40,641 (18.0) | 7,371 (18.0) | 12,045 (18.9) | 2,755 (19.3) | | < 0.001^‡^ |
| Craft and Related  Workers | 41,986 (14.9) | 7,844 (14.8) | 12,407 (15.1) | 3,986 (15.0) | < 0.001^‡^ | 35,250 (15.6) | 6,664 (16.3) | 10,003 (15.7) | 2,256 (15.8) | | < 0.001^‡^ |
| Plant & Machine  Operators and  Assemblers | 41,211 (14.7) | 7,690 (14.5) | 12,577 (15.3) | 4,265 (16.0) | < 0.001^‡^ | 33,889 (15.0) | 5,955 (14.5) | 9,874 (15.5) | 2,331 (16.4) | | < 0.001^‡^ |
| Elementary  Occupations | 32,577 (11.6) | 6,235 (11.7) | 9,769 (11.9) | 3,600 (13.5) | < 0.001^‡^ | 25,115 (11.1) | 4,702 (11.5) | 7,151 (11.2) | 1,781 (12.5) | | < 0.001^‡^ |
| Unemployed | 9,888 (3.5) | 1,952 (3.7) | 2,804 (3.4) | 1,013 (3.8) | < 0.001^‡^ | 6,877 (3.1) | 1,316 (3.2) | 2,002 (3.1) | 515 (3.6) | | < 0.001^‡^ |
| Occupation of Student's Mother, n (%) |  |  |  |  |  |  |  |  |  | |  |
| Missing value | 3,847 (1.4) | 724 (1.4) | 1,164 (1.4) | 423 (1.6) |  | 2,421 (1.1) | 501 (1.2) | 804 (1.3) | 205 (1.4) | |  |
| Managers and  Administrators | 8,397 (3.0) | 1,391 (2.6) | 2,567 (3.1) | 882 (3.3) | < 0.001^‡^ | 6,334 (2.8) | 1,000 (2.4) | 1,979 (3.1) | 413 (2.9) | | < 0.001^‡^ |
| Professionals | 5,728 (2.0) | 1,073 (2.0) | 1,419 (1.7) | 332 (1.2) | < 0.001^‡^ | 4,130 (1.8) | 741 (1.8) | 976 (1.5) | 161 (1.1) | | < 0.001^‡^ |
| Associate  Professionals | 13,177 (4.7) | 2,491 (4.7) | 3,500 (4.3) | 960 (3.6) | < 0.001^‡^ | 10,447 (4.6) | 1,869 (4.6) | 2,657 (4.2) | 534 (3.7) | | < 0.001^‡^ |
| Clerks | 47,327 (16.8) | 8,932 (16.8) | 13,646 (16.6) | 4,416 (16.6) | < 0.001^‡^ | 39,418 (17.5) | 7,021 (17.1) | 11,299 (17.7) | 2,614 (18.4) | | < 0.001^‡^ |
| Service Workers  and Shop Sales  Workers | 32,190 (11.5) | 5,554 (10.5) | 10,720 (13.0) | 4,067 (15.3) | < 0.001^‡^ | 23,039 (10.2) | 3,624 (8.8) | 7,591 (11.9) | 1,989 (14.0) | | < 0.001^‡^ |
| Craft and Related  Workers | 2,718 (1.0) | 456 (0.9) | 916 (1.1) | 331 (1.2) | < 0.001^‡^ | 2,244 (1.0) | 385 (0.9) | 734 (1.2) | 195 (1.4) | | < 0.001^‡^ |
| Plant & Machine  Operators and  Assemblers | 950 (0.3) | 147 (0.3) | 342 (0.4) | 133 (0.5) | < 0.001^‡^ | 766 (0.3) | 122 (0.3) | 236 (0.4) | 75 (0.5) | | < 0.001^‡^ |
| Elementary  Occupations | 10,876 (3.9) | 1,939 (3.7) | 3,514 (4.3) | 1,425 (5.3) | < 0.001^‡^ | 7,999 (3.6) | 1,399 (3.4) | 2,559 (4.0) | 713 (5.0) | | < 0.001^‡^ |
| Unemployed | 155,788 (55.4) | 30,393 (57.2) | 44,366 (54.0) | 13,677 (51.3) | < 0.001^‡^ | 128,496 (57.0) | 24,288 (59.3) | 34,862 (54.7) | 7,346 (51.6) | | < 0.001^‡^ |
| Type of Housing, n (%) |  |  |  |  |  |  |  |  |  | |  |
| Missing value | 4,846 (1.7) | 811 (1.5) | 1,376 (1.7) | 418 (1.6) |  | 3,781 (1.7) | 624 (1.5) | 1,103 (1.7) | 192 (1.3) | |  |
| Block-Self-  contained | 116,760 (41.6) | 22,064 (41.6) | 33,712 (41.0) | 10,163 (38.1) | < 0.001^‡^ | 96,013 (42.6) | 17,220 (42.1) | 26,657 (41.8) | 5,469 (38.4) | | < 0.001^‡^ |
| Block-Non Self-  contained | 6,386 (2.3) | 1,162 (2.2) | 1,938 (2.4) | 683 (2.6) | < 0.001^‡^ | 4,187 (1.9) | 741 (1.8) | 1,210 (1.9) | 323 (2.3) | | < 0.001^‡^ |
| Housing Authority  Home Ownership  Estate | 44,400 (15.8) | 8,539 (16.1) | 12,885 (15.7) | 4,163 (15.6) | < 0.001^‡^ | 37,844 (16.8) | 6,926 (16.9) | 10,845 (17.0) | 2,379 (16.7) | | < 0.001^‡^ |
| Housing  Authority/Society  Blocks | 95,611 (34.0) | 17,950 (33.8) | 28,450 (34.6) | 9,942 (37.3) | < 0.001^‡^ | 73,485 (32.6) | 13,488 (32.9) | 21,096 (33.1) | 5,189 (36.4) | | < 0.001^‡^ |
| Village Houses | 10,243 (3.6) | 2,054 (3.9) | 3,041 (3.7) | 1,042 (3.9) | < 0.001^‡^ | 7,670 (3.4) | 1,481 (3.6) | 2,149 (3.4) | 547 (3.8) | | < 0.001^‡^ |
| Institution | 2,752 (1.0) | 520 (1.0) | 752 (0.9) | 235 (0.9) | < 0.001^‡^ | 2,314 (1.0) | 470 (1.1) | 637 (1.0) | 146 (1.0) | | < 0.001^‡^ |
| ^‡^ Comparison among all groups with significance | | | | | | | | | |  |  |

| **Supple. Table S2** Baseline personal lifestyles and psychological wellbeing of children by weight status | | | | | | | | | | | | | |
| --- | --- | --- | --- | --- | --- | --- | --- | --- | --- | --- | --- | --- | --- |
|  | Primary Four Cohort (N1 = 442,898) | | | | | Primary Six Cohort (N2 = 344,186) | | | | | | | |
| Variables | Normal Weight (*n* = 280998) | Underweight (*n* = 53100) | Overweight (*n* = 82154) | Obese (*n* = 26646) | *p* Value | Normal Weight (*n* = 225,294) | Underweight (*n* = 40,950) | | Overweight (*n* = 63,697) | | Obese (*n* = 14,245) | | *p* Value |
| Breakfast Eating Habit, n (%) |  |  |  |  |  |  |  | |  | |  | |  |
| Missing value | 283 (0.1) | 45 (0.1) | 69 (0.1) | 33 (0.1) |  | 177 (0.1) | 26 (0.1) | | 76 (0.1) | | 15 (0.1) | |  |
| home | 247,611 (88.1) | 47,572 (89.6) | 69,413 (84.5) | 21,145 (79.4) | < 0.001^‡^ | 192,513 (85.4) | 35,991 (87.9) | | 51,280 (80.5) | | 10,735 (75.4) | | < 0.001^‡^ |
| rarely at home | 21,053 (7.5) | 3,635 (6.8) | 7,468 (9.1) | 3,165 (11.8) | < 0.001^‡^ | 19,323 (8.6) | 3,159 (7.7) | | 6,748 (10.6) | | 1,868 (13.1) | | < 0.001^‡^ |
| no breakfast | 12,051 (4.3) | 1,848 (3.5) | 5,204 (6.3) | 2,303 (8.6) | < 0.001^‡^ | 13,281 (5.9) | 1,774 (4.3) | | 5,593 (8.8) | | 1,627 (11.4) | | < 0.001^‡^ |
| Sweetness Preference during Past 7 days, n (%) |  |  |  |  |  |  |  | |  | |  | |  |
| Missing value | 674 (0.2) | 129 (0.2) | 214 (0.3) | 81 (0.3) |  | 402 (0.2) | 66 (0.2) | | 108 (0.2) | | 30 (0.2) | |  |
| 0-3 times | 92,655 (33.0) | 18,533 (34.9) | 25,493 (31.0) | 8,464 (31.8) | < 0.001^‡^ | 69,450 (30.8) | 13,581 (33.2) | | 17,169 (27.0) | | 3,628 (25.5) | | < 0.001^‡^ |
| 4-6 times | 160,588 (57.1) | 29,967 (56.4) | 47,400 (57.7) | 15,017 (56.4) | < 0.001^‡^ | 140,912 (62.5) | 24,944 (60.9) | | 41,119 (64.6) | | 9.202 (64.6) | | < 0.001^‡^ |
| once daily | 18,414 (6.6) | 3,078 (5.8) | 6,043 (7.4) | 2,010 (7.5) | < 0.001^‡^ | 10,256 (4.6) | 1,695 (4.1) | | 3,699 (5.8) | | 934 (6.6) | | < 0.001^‡^ |
| 2 times or above  daily | 8,667 (3.1) | 1,393 (2.6) | 3,004 (3.7) | 1,070 (4.0) | < 0.001^‡^ | 4,274 (1.9) | 664 (1.6) | | 1,602 (2.5) | | 451 (3.2) | | < 0.001^‡^ |
| Junk Food Intake Habits, n (%) |  |  |  |  |  |  |  | |  | |  | |  |
| Missing value | 705 (0.3) | 119 (0.2) | 223 (0.3) | 95 (0.4) |  | 441 (0.2) | 76 (0.2) | | 141 (0.2) | | 25 (0.2) | |  |
| Everyday | 15,053 (5.4) | 3104 (5.8) | 3,933 (4.8) | 1,255 (4.7) | < 0.001^‡^ | 13,466 (6.0) | 2,792 (6.8) | | 2,706 (4.2) | | 526 (3.7) | | < 0.001^‡^ |
| Occasionally | 170,541 (60.7) | 33,405 (62.9) | 48,632 (59.2) | 15,284 (57.4) | < 0.001^‡^ | 145,487 (64.6) | 26,967 (65.9) | | 39,423 (61.9) | | 8,385 (58.9) | | < 0.001^‡^ |
| Rarely | 90,609 (32.2) | 15,838 (29.8) | 27,881 (33.9) | 9,424 (35.4) | < 0.001^‡^ | 63,656 (28.3) | 10,784 (26.3) | | 20,539 (32.2) | | 5,034 (35.3) | | < 0.001^‡^ |
| Never | 4,090 (1.5) | 634 (1.2) | 1,485 (1.8) | 588 (2.2) | < 0.001^‡^ | 2,244 (1.0) | | 331 (0.8) | | 888 (1.4) | | 275 (1.9) | < 0.001^‡^ |
| Fruit/ vegetable Intake, n (%) |  |  |  |  |  |  | |  | |  | |  |  |
| Missing value | 11,229 (4.0) | 2105 (4.0) | 3627 (4.4) | 1,229 (4.6) |  | 10,593 (4.7) | | 1,725 (4.2) | | 3,263 (5.1) | | 792 (5.6) |  |
| at least thrice a  day | 71,618 (25.5) | 12,680 (23.9) | 20,888 (25.4) | 6,454 (24.2) | < 0.001^‡^ | 48,730 (21.6) | | 8,161 (19.9) | | 14,119 (22.2) | | 3,080 (21.6) | < 0.001^‡^ |
| once or twice a  day | 152,555 (54.3) | 29,240 (55.1) | 44,003 (53.6) | 14,041 (52.7) | < 0.001^‡^ | 128,899 (57.2) | | 23,626 (57.7) | | 35,955 (56.4) | | 7,868 (55.2) | < 0.001^‡^ |
| once every few  days | 35,593 (12.7) | 7,228 (13.6) | 10,503 (12.8) | 3,676 (13.8) | < 0.001^‡^ | 31,280 (13.9) | | 6,357 (15.5) | | 8,639 (13.6) | | 2,035 (14.3) | < 0.001^‡^ |
| less than once a  week | 10,003 (3.6) | 1,847 (3.5) | 3,133 (3.8) | 1,246 (4.7) | < 0.001^‡^ | 5,792 (2.6) | | 1,081 (2.6) | | 1,721 (2.7) | | 470 (3.3) | < 0.001^‡^ |
| Milk Consumption Habit, n (%) |  |  |  |  |  |  | |  | |  | |  |  |
| Missing value | 904 (0.3) | 171 (0.3) | 289 (0.4) | 95 (0.4) |  | 504 (0.2) | | 91 (0.2) | | 153 (0.2) | | 47 (0.3) |  |
| at least once a  day | 93,499 (33.3) | 17,602 (33.1) | 22,408 (27.3) | 5,971 (22.4) | < 0.001^‡^ | 61,479 (27.3) | | 11,701 (28.6) | | 13,814 (21.7) | | 2,470 (17.3) | < 0.001^‡^ |
| once every few  days | 82,500 (29.4) | 15,780 (29.7) | 24,099 (29.3) | 7,632 (28.6) | < 0.001^‡^ | 67,828 (30.1) | | 12,534 (30.6) | | 18,790 (29.5) | | 4,069 (28.6) | < 0.001^‡^ |
| less than once a  week | 51,716 (18.4) | 9,689 (18.2) | 17,164 (20.9) | 6,906 (22.9) | < 0.001^‡^ | 50,745 (22.5) | | 8,802 (21.5) | | 15,917 (25.0) | | 3,808 (26.7) | < 0.001^‡^ |
| Never | 52,379 (18.6) | 9,858 (18.6) | 18,194 (22.1) | 6,852 (25.7) | < 0.001^‡^ | 44,738 (19.9) | | 7,822 (19.1) | | 15,023 (23.6) | | 3,851 (27.0) | < 0.001^‡^ |
| Frequency of Aerobic Exercise, n (%) |  |  |  |  |  |  | |  | |  | |  |  |
| Missing value | 4,401 (1.6) | 824 (1.6) | 1,384 (1.7) | 487 (1.8) |  | 2,955 (0.3) | | 527 (1.3) | | 983 (1.5) | | 227 (1.6) |  |
| at least thrice a  day | 89,566 (31.9) | 14,729 (27.7) | 22,508 (27.4) | 6,496 (24.4) | < 0.001^‡^ | 66,401 (29.5) | | 10,213 (24.9) | | 15,886 (24.9) | | 3,165 (22.2) | < 0.001^‡^ |
| once or twice a  day | 125,063 (44.5) | 24,244 (45.7) | 38,257 (46.6) | 12,063 (45.3) | < 0.001^‡^ | 104,915 (46.6) | | 19,463 (47.5) | | 31,025 (48.7) | | 6,806 (47.8) | < 0.001^‡^ |
| once every few  days | 44,152 (15.7) | 9,315 (17.5) | 14,530 (17.7) | 5,433 (20.4) | < 0.001^‡^ | 38,613 (17.1) | | 8,031 (19.6) | | 12,163 (19.1) | | 3,077 (21.6) | < 0.001^‡^ |
| less than once a  week | 17,816 (6.3) | 3,988 (7.5) | 5,475 (6.7) | 2,167 (8.1) | < 0.001^‡^ | 12,410 (5.5) | | 2,716 (6.6) | | 3,640 (5.7) | | 970 (6.8) | < 0.001^‡^ |
| Hours of Aerobic Exercise, n (%) |  |  |  |  |  |  | |  | |  | |  |  |
| Missing value | 4,434 (1.6) | 1,413 (1.7) | 893 (1.7) | 478 (1.8) |  | 3,153 (1.4) | | 616 (1.5) | | 1,033 (1.6) | | 245 (1.7) |  |
| more than an  hour | 92,537 (32.9) | 26,928 (32.8) | 14,695 (27.7) | 8,572 (32.2) | < 0.001^‡^ | 89,486 (39.7) | | 13,746 (33.6) | | 24,247 (38.1) | | 5,109 (35.9) | < 0.001^‡^ |
| half to one hour | 111,469 (39.7) | 32,151 (39.1) | 21,818 (41.1) | 9,963 (37.4) | < 0.001^‡^ | 81,092 (36.0) | | 15,585 (38.1) | | 23,278 (36.5) | | 5,204 (36.5) | < 0.001^‡^ |
| less than half an  hour | 51,992 (18.5) | 15,423 (18.8) | 11,128 (21.0) | 5,172 (19.4) | < 0.001^‡^ | 37,293 (16.6) | | 7,933 (19.4) | | 10,975 (17.2) | | 2,590 (18.2) | < 0.001^‡^ |
| zero | 20,566 (7.3) | 6,239 (7.6) | 4,566 (8.6) | 2,461 (9.2) | < 0.001^‡^ | 14,270 (6.3) | | 3,070 (7.5) | | 4,164 (6.5) | | 1,097 (7.7) | < 0.001^‡^ |
| Daily Hours of TV Viewing, n (%) |  |  |  |  |  |  | |  | |  | |  |  |
| Missing value | 3,181 (1.1) | 553 (1.0) | 1,096 (1.3) | 368 (1.4) |  | 2,412 (1.1) | | 436 (1.1) | | 758 (1.2) | | 202 (1.4) |  |
| less than an hour | 60,103 (21.4) | 11,073 (20.9) | 14,960 (18.2) | 3,814 (14.3) | < 0.001^‡^ | 33,894 (15.0) | | 5,998 (14.6) | | 8,150 (12.8) | | 1,435 (10.1) | < 0.001^‡^ |
| one or two hours | 112,788 (40.1) | 21,658 (40.8) | 31,807 (38.7) | 9,617 (36.1) | < 0.001^‡^ | 87,460 (38.8) | | 16,049 (39.2) | | 23,295 (36.6) | | 4,690 (32.9) | < 0.001^‡^ |
| two to four hours | 73,959 (26.3) | 14,271 (26.9) | 23,204 (28.2) | 8,254 (31.0) | < 0.001^‡^ | 73,813 (32.8) | | 13,742 (33.6) | | 22,087 (34.7) | | 5,207 (36.6) | < 0.001^‡^ |
| more than four  hours | 30,967 (11.0) | 5,454 (10.4) | 11,087 (13.5) | 4,593 (17.2) | < 0.001^‡^ | 27,715 (12.3) | | 4,725 (11.5) | | 9,407 (14.8) | | 2,711 (19.0) | < 0.001^‡^ |
| SEI Score a, mean (SD) |  |  |  |  |  |  | |  | |  | |  |  |
| Total | 37.33 (6.82) | 37.26 (6.75) ^*^ | 36.67 (7.05) ^†^ | 35.59 (7.22) ^†^ | < 0.05^‡^ | 38.13 (6.84) | | 38.06 (6.75) | | 37.18 (7.26) ^†^ | | 36.12 (7.41) ^†^ | < 0.001^§^ |
| General | 15.00 (3.20) | 15.05 (3.19) ^*^ | 14.69 (3.31) ^†^ | 14.22 (3.39) ^†^ | < 0.05^‡^ | 15.43 (3.19) | | 15.52 (3.16) | | 14.97 (3.38) | | 14.56 (3.43) | < 0.001^‡^ |
| Social | 6.58 (1.80) | 6.35 (1.80) | 6.46 (1.83) | 6.22 (1.86) | < 0.001^‡^ | 6.97 (1.77) | | 6.64 (1.77) | | 6.77 (1.86) | | 6.54 (1.87) | < 0.001^‡^ |
| School-related | 7.14 (1.81) | 7.18 (1.78) | 7.02 (1.81) | 6.80 (1.89) | < 0.001^‡^ | 7.13 (1.88) | | 7.19 (1.85) | | 6.97 (1.94) | | 6.71 (2.00) | < 0.001^‡^ |
| Parent-related | 8.61 (1.84) | 8.68 (1.80) | 8.50 (1.90) | 8.35 (1.98) | < 0.001¶ | 8.61 (1.90) | | 8.72 (1.82) | | 8.48 (2.00) | | 8.31 (2.10) | < 0.001^‡^ |
| RBQ Score b, mean (SD) |  |  |  |  |  |  | |  | |  | |  |  |
| Total | 9.30 (5.80) | 9.35 (5.82) | 9.34 (5.80) | 9.71 (5.90) ^†^ | < 0.001^§^ | 9.33 (5.44) | | 9.57 (5.48) | | 9.62 (5.56) | | 9.98 (5.72) | < 0.001^‡^ |
| Conduct | 1.51 (1.30) | 1.42 (1.27) ^†^ | 1.51 (1.30) | 1.56 (1.32) ^†^ | < 0.001^§^ | 1.39 (1.24) | | 1.32 (1.21) | | 1.45 (1.27) | | 1.52 (1.32) | < 0.001^‡^ |
| Emotion | 1.41 (1.26) | 1.53 (1.30) | 1.33 (1.23) | 1.30 (1.23) | < 0.001^‡^ | 1.42 (1.24) | | 1.54 (1.28) | | 1.33 (1.22) | | 1.29 (1.23) | < 0.001^‡^ |
| Hyperactivity | 1.76 (1.56) | 1.63 (1.54) ^†^ | 1.77 (1.55) ^*^ | 1.78 (1.54) ^*^ | < 0.05^‡^ | 1.34 (1.44) | | 1.29 (1.43) | | 1.41 (1.45) | | 1.42 (1.46) | < 0.001^‡^ |
| *SD* standard deviation*, SEI* Culture Free Self-Esteem Inventory for Children Questionnaire, *RBQ* Rutter Behaviour Questionnaire. | | | | | | | | | | | | | |
| ^‡^ Comparison among all groups with significance | | | | | |  |  | |  | |  | |  |
| ^§^ Comparison between the groups with significance | | | | |  |  |  | |  | |  | |  |
| ^*^ P < 0.05, when comparison with normal weight group | | | | |  |  |  | |  | |  | |  |
| ^†^ P < 0.001, when comparison with normal weight group | | | | |  |  |  | |  | |  | |  |

| **Supple. Table S3** Prediction accuracy of different machine learning models. | | | | | | | |
| --- | --- | --- | --- | --- | --- | --- | --- |
| (a) based on primary four cohort | | | | | | | |
| Prediction Window | Prediction Performances | Random Forest | XG Boost | Decision Tree | k-NN | Logistic Regression | Support Vector Machine |
| Primary Five | Accuracy | 0.835 | 0.845 | 0.777 | 0.709 | 0.734 | 0.740 |
|  | Micro-averaging AUC | 0.963 | 0.972 | 0.850 | 0.867 | 0.884 | 0.886 |
|  | Macro-averaging AUC | 0.945 | 0.960 | 0.810 | 0.870 | 0.874 | 0.875 |
| Primary Six | Accuracy | 0.794 | 0.806 | 0.723 | 0.655 | 0.699 | 0.714 |
|  | Micro-averaging AUC | 0.946 | 0.958 | 0.815 | 0.842 | 0.863 | 0.878 |
|  | Macro-averaging AUC | 0.915 | 0.937 | 0.760 | 0.854 | 0.863 | 0.878 |
| Secondary One | Accuracy | 0.774 | 0.786 | 0.699 | 0.605 | 0.654 | 0.669 |
|  | Micro-averaging AUC | 0.938 | 0.950 | 0.799 | 0.803 | 0.844 | 0.859 |
|  | Macro-averaging AUC | 0.890 | 0.915 | 0.719 | 0.795 | 0.851 | 0.864 |
| Secondary Two | Accuracy | 0.761 | 0.774 | 0.680 | 0.561 | 0.618 | 0.631 |
|  | Micro-averaging AUC | 0.930 | 0.944 | 0.786 | 0.782 | 0.833 | 0.846 |
|  | Macro-averaging AUC | 0.862 | 0.894 | 0.691 | 0.796 | 0.841 | 0.854 |
| Secondary Three | Accuracy | 0.750 | 0.761 | 0.668 | 0.533 | 0.593 | 0.607 |
|  | Micro-averaging AUC | 0.924 | 0.939 | 0.779 | 0.762 | 0.820 | 0.834 |
|  | Macro-averaging AUC | 0.841 | 0.876 | 0.667 | 0.773 | 0.825 | 0.839 |
| Secondary Four | Accuracy | 0.742 | 0.751 | 0.653 | 0.516 | 0.569 | 0.581 |
|  | Micro-averaging AUC | 0.919 | 0.932 | 0.769 | 0.750 | 0.811 | 0.823 |
|  | Macro-averaging AUC | 0.823 | 0.859 | 0.657 | 0.754 | 0.811 | 0.823 |
| Secondary Five | Accuracy | 0.727 | 0.742 | 0.638 | 0.512 | 0.546 | 0.558 |
|  | Micro-averaging AUC | 0.912 | 0.926 | 0.759 | 0.741 | 0.802 | 0.814 |
|  | Macro-averaging AUC | 0.804 | 0.846 | 0.630 | 0.704 | 0.793 | 0.805 |
| Secondary Six | Accuracy | 0.707 | 0.722 | 0.618 | 0.501 | 0.555 | 0.568 |
|  | Micro-averaging AUC | 0.901 | 0.916 | 0.747 | 0.739 | 0.799 | 0.812 |
|  | Macro-averaging AUC | 0.779 | 0.834 | 0.626 | 0.687 | 0.773 | 0.786 |
| (b) based on primary six cohort | | | | | | | |
| Prediction Window | Prediction Performances | Random Forest | XG Boost | Decision Tree | k-NN | Logistic Regression | Supportive Vector Machine |
| Secondary One | Accuracy | 0.840 | 0.847 | 0.780 | 0.723 | 0.738 | 0.753 |
|  | Micro-averaging AUC | 0.963 | 0.973 | 0.855 | 0.872 | 0.876 | 0.891 |
|  | Macro-averaging AUC | 0.938 | 0.957 | 0.797 | 0.866 | 0.875 | 0.888 |
| Secondary Two | Accuracy | 0.802 | 0.814 | 0.736 | 0.659 | 0.685 | 0.698 |
|  | Micro-averaging AUC | 0.950 | 0.961 | 0.824 | 0.834 | 0.857 | 0.870 |
|  | Macro-averaging AUC | 0.905 | 0.928 | 0.736 | 0.816 | 0.861 | 0.874 |
| Secondary Three | Accuracy | 0.782 | 0.792 | 0.705 | 0.616 | 0.643 | 0.657 |
|  | Micro-averaging AUC | 0.940 | 0.952 | 0.807 | 0.808 | 0.842 | 0.856 |
|  | Macro-averaging AUC | 0.877 | 0.907 | 0.706 | 0.785 | 0.846 | 0.860 |
| Secondary Four | Accuracy | 0.766 | 0.777 | 0.687 | 0.581 | 0.620 | 0.632 |
|  | Micro-averaging AUC | 0.933 | 0.946 | 0.792 | 0.787 | 0.832 | 0.844 |
|  | Macro-averaging AUC | 0.856 | 0.890 | 0.672 | 0.755 | 0.829 | 0.841 |
| Secondary Five | Accuracy | 0.752 | 0.763 | 0.668 | 0.557 | 0.590 | 0.602 |
|  | Micro-averaging AUC | 0.925 | 0.938 | 0.778 | 0.775 | 0.823 | 0.835 |
|  | Macro-averaging AUC | 0.841 | 0.876 | 0.659 | 0.743 | 0.807 | 0.819 |
| Secondary Six | Accuracy | 0.721 | 0.740 | 0.640 | 0.548 | 0.587 | 0.600 |
|  | Micro-averaging AUC | 0.911 | 0.927 | 0.765 | 0.761 | 0.814 | 0.827 |
|  | Macro-averaging AUC | 0.810 | 0.857 | 0.716 | 0.641 | 0.784 | 0.797 |
| XG Boost eXtreme Gradient Boosting; k-NN K-nearest Neighbors, AUC area under the curve. | | | | | | | |

| **Supple. Table S4** Prediction performances of different machine learning models by weight status | | | | | | | | | | | | | | | | | | | |
| --- | --- | --- | --- | --- | --- | --- | --- | --- | --- | --- | --- | --- | --- | --- | --- | --- | --- | --- | --- |
| Prediction Model | | Random Forest | | | XG Boost | | | Decision Tree | | | k-Nearest Neighbors | | | Logistic Regression | | | Support Vector Machine | | |
| Prediction Performance | | Precision | Recall | F1-score | Precision | Recall | F1-score | Precision | Recall | F1-score | Precision | Recall | F1-score | Precision | Recall | F1-score | Precision | Recall | F1-score |
|  |  | Primary Four Prediction Cohort | | | | | | | | | | | | | | | | | |
| Primary Five | Normal | 0.878 | 0.907 | 0.892 | 0.894 | 0.901 | 0.898 | 0.861 | 0.838 | 0.849 | 0.907 | 0.674 | 0.773 | 0.918 | 0.705 | 0.797 | 0.848 | 0.877 | 0.862 |
|  | Underweight | 0.750 | 0.679 | 0.713 | 0.757 | 0.727 | 0.742 | 0.614 | 0.663 | 0.638 | 0.455 | 0.829 | 0.588 | 0.485 | 0.879 | 0.625 | 0.725 | 0.654 | 0.688 |
|  | Overweight | 0.749 | 0.725 | 0.737 | 0.754 | 0.753 | 0.754 | 0.655 | 0.661 | 0.658 | 0.584 | 0.743 | 0.654 | 0.618 | 0.718 | 0.664 | 0.729 | 0.705 | 0.717 |
|  | Obese | 0.773 | 0.723 | 0.747 | 0.763 | 0.767 | 0.765 | 0.658 | 0.719 | 0.687 | 0.593 | 0.739 | 0.658 | 0.595 | 0.817 | 0.688 | 0.723 | 0.673 | 0.697 |
| Primary Six | Normal | 0.848 | 0.890 | 0.868 | 0.866 | 0.882 | 0.874 | 0.833 | 0.797 | 0.815 | 0.888 | 0.614 | 0.726 | 0.900 | 0.678 | 0.774 | 0.818 | 0.860 | 0.838 |
|  | Underweight | 0.689 | 0.576 | 0.627 | 0.693 | 0.638 | 0.664 | 0.525 | 0.594 | 0.557 | 0.395 | 0.821 | 0.534 | 0.450 | 0.860 | 0.591 | 0.664 | 0.551 | 0.602 |
|  | Overweight | 0.676 | 0.633 | 0.654 | 0.687 | 0.671 | 0.679 | 0.559 | 0.564 | 0.561 | 0.519 | 0.670 | 0.585 | 0.557 | 0.652 | 0.601 | 0.656 | 0.613 | 0.634 |
|  | Obese | 0.671 | 0.641 | 0.655 | 0.671 | 0.702 | 0.686 | 0.513 | 0.637 | 0.568 | 0.498 | 0.772 | 0.605 | 0.501 | 0.785 | 0.611 | 0.621 | 0.591 | 0.605 |
| Secondary One | Normal | 0.835 | 0.892 | 0.863 | 0.852 | 0.885 | 0.868 | 0.825 | 0.784 | 0.804 | 0.872 | 0.578 | 0.695 | 0.904 | 0.626 | 0.740 | 0.805 | 0.862 | 0.833 |
|  | Underweight | 0.642 | 0.473 | 0.545 | 0.658 | 0.536 | 0.591 | 0.454 | 0.520 | 0.485 | 0.345 | 0.748 | 0.472 | 0.385 | 0.857 | 0.531 | 0.617 | 0.448 | 0.520 |
|  | Overweight | 0.592 | 0.544 | 0.567 | 0.607 | 0.585 | 0.596 | 0.475 | 0.501 | 0.488 | 0.416 | 0.625 | 0.499 | 0.470 | 0.615 | 0.533 | 0.572 | 0.524 | 0.547 |
|  | Obese | 0.589 | 0.516 | 0.550 | 0.605 | 0.593 | 0.599 | 0.444 | 0.551 | 0.492 | 0.377 | 0.599 | 0.463 | 0.430 | 0.757 | 0.548 | 0.539 | 0.466 | 0.500 |
| Secondary Two | Normal | 0.826 | 0.894 | 0.859 | 0.838 | 0.894 | 0.865 | 0.822 | 0.771 | 0.796 | 0.874 | 0.521 | 0.653 | 0.898 | 0.591 | 0.712 | 0.796 | 0.864 | 0.829 |
|  | Underweight | 0.600 | 0.403 | 0.482 | 0.627 | 0.459 | 0.530 | 0.406 | 0.475 | 0.438 | 0.301 | 0.777 | 0.433 | 0.341 | 0.842 | 0.485 | 0.575 | 0.378 | 0.457 |
|  | Overweight | 0.535 | 0.451 | 0.489 | 0.558 | 0.471 | 0.511 | 0.407 | 0.433 | 0.420 | 0.365 | 0.574 | 0.446 | 0.416 | 0.565 | 0.479 | 0.515 | 0.431 | 0.469 |
|  | Obese | 0.487 | 0.487 | 0.487 | 0.507 | 0.570 | 0.536 | 0.348 | 0.513 | 0.415 | 0.336 | 0.666 | 0.446 | 0.372 | 0.738 | 0.495 | 0.437 | 0.437 | 0.437 |
| Secondary Three | Normal | 0.813 | 0.896 | 0.852 | 0.822 | 0.898 | 0.859 | 0.814 | 0.758 | 0.785 | 0.862 | 0.490 | 0.624 | 0.888 | 0.562 | 0.688 | 0.783 | 0.866 | 0.822 |
|  | Underweight | 0.594 | 0.366 | 0.453 | 0.624 | 0.425 | 0.505 | 0.399 | 0.465 | 0.429 | 0.307 | 0.759 | 0.437 | 0.343 | 0.824 | 0.484 | 0.569 | 0.341 | 0.428 |
|  | Overweight | 0.473 | 0.378 | 0.420 | 0.492 | 0.371 | 0.423 | 0.364 | 0.408 | 0.384 | 0.310 | 0.545 | 0.396 | 0.360 | 0.522 | 0.426 | 0.453 | 0.358 | 0.400 |
|  | Obese | 0.416 | 0.404 | 0.410 | 0.428 | 0.479 | 0.452 | 0.298 | 0.436 | 0.354 | 0.277 | 0.617 | 0.383 | 0.315 | 0.712 | 0.437 | 0.366 | 0.354 | 0.360 |
| Secondary Four | Normal | 0.802 | 0.894 | 0.846 | 0.807 | 0.900 | 0.851 | 0.804 | 0.746 | 0.774 | 0.850 | 0.469 | 0.605 | 0.878 | 0.532 | 0.663 | 0.772 | 0.864 | 0.816 |
|  | Underweight | 0.580 | 0.367 | 0.449 | 0.598 | 0.416 | 0.491 | 0.390 | 0.461 | 0.423 | 0.314 | 0.743 | 0.441 | 0.346 | 0.812 | 0.485 | 0.555 | 0.342 | 0.424 |
|  | Overweight | 0.445 | 0.331 | 0.380 | 0.461 | 0.290 | 0.356 | 0.327 | 0.365 | 0.345 | 0.285 | 0.531 | 0.371 | 0.322 | 0.500 | 0.391 | 0.425 | 0.311 | 0.360 |
|  | Obese | 0.342 | 0.297 | 0.318 | 0.386 | 0.421 | 0.403 | 0.241 | 0.378 | 0.294 | 0.226 | 0.564 | 0.322 | 0.265 | 0.679 | 0.381 | 0.292 | 0.247 | 0.268 |
| Secondary Five | Normal | 0.781 | 0.886 | 0.830 | 0.786 | 0.898 | 0.839 | 0.779 | 0.728 | 0.753 | 0.809 | 0.474 | 0.598 | 0.864 | 0.488 | 0.624 | 0.751 | 0.856 | 0.800 |
|  | Underweight | 0.606 | 0.394 | 0.477 | 0.631 | 0.449 | 0.524 | 0.424 | 0.471 | 0.447 | 0.358 | 0.686 | 0.470 | 0.386 | 0.812 | 0.523 | 0.581 | 0.369 | 0.452 |
|  | Overweight | 0.402 | 0.296 | 0.341 | 0.429 | 0.234 | 0.303 | 0.298 | 0.352 | 0.322 | 0.248 | 0.501 | 0.332 | 0.272 | 0.476 | 0.346 | 0.382 | 0.276 | 0.321 |
|  | Obese | 0.290 | 0.170 | 0.215 | 0.415 | 0.310 | 0.355 | 0.215 | 0.310 | 0.254 | 0.163 | 0.420 | 0.234 | 0.216 | 0.675 | 0.327 | 0.240 | 0.120 | 0.165 |
| Secondary Six | Normal | 0.755 | 0.871 | 0.809 | 0.765 | 0.881 | 0.819 | 0.756 | 0.708 | 0.731 | 0.778 | 0.455 | 0.574 | 0.848 | 0.489 | 0.620 | 0.725 | 0.841 | 0.779 |
|  | Underweight | 0.602 | 0.424 | 0.498 | 0.630 | 0.488 | 0.550 | 0.456 | 0.497 | 0.476 | 0.395 | 0.684 | 0.501 | 0.437 | 0.807 | 0.567 | 0.577 | 0.399 | 0.473 |
|  | Overweight | 0.398 | 0.250 | 0.307 | 0.394 | 0.181 | 0.248 | 0.267 | 0.309 | 0.286 | 0.224 | 0.465 | 0.302 | 0.269 | 0.473 | 0.343 | 0.378 | 0.230 | 0.287 |
|  | Obese | 0.295 | 0.149 | 0.198 | 0.284 | 0.189 | 0.227 | 0.135 | 0.203 | 0.162 | 0.135 | 0.369 | 0.198 | 0.185 | 0.635 | 0.286 | 0.245 | 0.099 | 0.148 |
|  |  | Primary Six Prediction Cohort | | | | | | | | | | | | | | | | | |
| Secondary One | Normal | 0.887 | 0.916 | 0.901 | 0.902 | 0.906 | 0.904 | 0.874 | 0.844 | 0.859 | 0.922 | 0.704 | 0.798 | 0.935 | 0.715 | 0.810 | 0.857 | 0.886 | 0.871 |
|  | Underweight | 0.719 | 0.698 | 0.708 | 0.722 | 0.725 | 0.723 | 0.611 | 0.629 | 0.620 | 0.547 | 0.740 | 0.629 | 0.569 | 0.720 | 0.636 | 0.694 | 0.673 | 0.683 |
|  | Overweight | 0.743 | 0.645 | 0.691 | 0.738 | 0.700 | 0.718 | 0.581 | 0.649 | 0.613 | 0.451 | 0.825 | 0.584 | 0.466 | 0.881 | 0.610 | 0.723 | 0.625 | 0.671 |
|  | Obese | 0.722 | 0.655 | 0.687 | 0.701 | 0.729 | 0.715 | 0.573 | 0.661 | 0.614 | 0.529 | 0.718 | 0.609 | 0.537 | 0.836 | 0.654 | 0.672 | 0.605 | 0.637 |
| Secondary Two | Normal | 0.858 | 0.907 | 0.882 | 0.872 | 0.903 | 0.887 | 0.850 | 0.818 | 0.834 | 0.898 | 0.644 | 0.750 | 0.925 | 0.658 | 0.769 | 0.828 | 0.877 | 0.852 |
|  | Underweight | 0.611 | 0.561 | 0.585 | 0.632 | 0.599 | 0.615 | 0.497 | 0.514 | 0.506 | 0.433 | 0.655 | 0.522 | 0.483 | 0.658 | 0.557 | 0.586 | 0.536 | 0.560 |
|  | Overweight | 0.685 | 0.530 | 0.598 | 0.694 | 0.585 | 0.635 | 0.496 | 0.563 | 0.527 | 0.389 | 0.771 | 0.517 | 0.399 | 0.866 | 0.547 | 0.665 | 0.510 | 0.578 |
|  | Obese | 0.594 | 0.528 | 0.559 | 0.613 | 0.615 | 0.614 | 0.445 | 0.545 | 0.490 | 0.394 | 0.614 | 0.480 | 0.452 | 0.792 | 0.575 | 0.544 | 0.478 | 0.509 |
| Secondary Three | Normal | 0.839 | 0.903 | 0.870 | 0.853 | 0.899 | 0.875 | 0.836 | 0.788 | 0.811 | 0.887 | 0.594 | 0.712 | 0.919 | 0.606 | 0.731 | 0.809 | 0.873 | 0.840 |
|  | Underweight | 0.539 | 0.476 | 0.506 | 0.555 | 0.495 | 0.523 | 0.414 | 0.465 | 0.438 | 0.361 | 0.611 | 0.454 | 0.402 | 0.629 | 0.491 | 0.514 | 0.451 | 0.481 |
|  | Overweight | 0.647 | 0.463 | 0.540 | 0.666 | 0.527 | 0.589 | 0.453 | 0.519 | 0.484 | 0.369 | 0.759 | 0.497 | 0.385 | 0.853 | 0.530 | 0.627 | 0.443 | 0.520 |
|  | Obese | 0.550 | 0.435 | 0.486 | 0.536 | 0.536 | 0.536 | 0.360 | 0.461 | 0.404 | 0.312 | 0.554 | 0.399 | 0.388 | 0.753 | 0.512 | 0.500 | 0.385 | 0.436 |
| Secondary Four | Normal | 0.826 | 0.895 | 0.859 | 0.834 | 0.899 | 0.866 | 0.823 | 0.771 | 0.796 | 0.865 | 0.558 | 0.679 | 0.908 | 0.581 | 0.709 | 0.796 | 0.865 | 0.829 |
|  | Underweight | 0.484 | 0.412 | 0.445 | 0.499 | 0.390 | 0.438 | 0.362 | 0.414 | 0.386 | 0.304 | 0.561 | 0.394 | 0.348 | 0.583 | 0.436 | 0.459 | 0.387 | 0.420 |
|  | Overweight | 0.623 | 0.443 | 0.518 | 0.653 | 0.499 | 0.566 | 0.446 | 0.517 | 0.479 | 0.364 | 0.733 | 0.487 | 0.392 | 0.839 | 0.534 | 0.603 | 0.423 | 0.498 |
|  | Obese | 0.427 | 0.359 | 0.390 | 0.447 | 0.459 | 0.453 | 0.296 | 0.403 | 0.341 | 0.248 | 0.493 | 0.330 | 0.325 | 0.715 | 0.447 | 0.377 | 0.309 | 0.340 |
| Secondary Five | Normal | 0.806 | 0.889 | 0.845 | 0.814 | 0.893 | 0.851 | 0.803 | 0.750 | 0.776 | 0.840 | 0.518 | 0.641 | 0.896 | 0.531 | 0.667 | 0.776 | 0.859 | 0.815 |
|  | Underweight | 0.444 | 0.360 | 0.398 | 0.464 | 0.331 | 0.387 | 0.324 | 0.408 | 0.361 | 0.263 | 0.545 | 0.355 | 0.290 | 0.565 | 0.384 | 0.419 | 0.335 | 0.373 |
|  | Overweight | 0.643 | 0.462 | 0.538 | 0.660 | 0.511 | 0.576 | 0.468 | 0.509 | 0.488 | 0.393 | 0.730 | 0.511 | 0.422 | 0.836 | 0.561 | 0.623 | 0.442 | 0.518 |
|  | Obese | 0.409 | 0.274 | 0.328 | 0.476 | 0.418 | 0.445 | 0.275 | 0.371 | 0.316 | 0.232 | 0.485 | 0.314 | 0.278 | 0.694 | 0.397 | 0.359 | 0.224 | 0.278 |
| Secondary Six | Normal | 0.779 | 0.864 | 0.819 | 0.793 | 0.871 | 0.830 | 0.779 | 0.717 | 0.747 | 0.815 | 0.507 | 0.625 | 0.863 | 0.533 | 0.659 | 0.749 | 0.834 | 0.789 |
|  | Underweight | 0.374 | 0.317 | 0.343 | 0.395 | 0.265 | 0.317 | 0.283 | 0.366 | 0.319 | 0.240 | 0.510 | 0.326 | 0.258 | 0.485 | 0.337 | 0.349 | 0.292 | 0.318 |
|  | Overweight | 0.623 | 0.460 | 0.529 | 0.646 | 0.536 | 0.586 | 0.474 | 0.528 | 0.500 | 0.425 | 0.719 | 0.534 | 0.458 | 0.804 | 0.584 | 0.603 | 0.440 | 0.509 |
|  | Obese | 0.320 | 0.170 | 0.222 | 0.385 | 0.278 | 0.323 | 0.191 | 0.257 | 0.220 | 0.173 | 0.386 | 0.239 | 0.244 | 0.693 | 0.361 | 0.270 | 0.120 | 0.172 |

XG Boost eXtreme Gradient Boosting

The precision, recall, F1-score, and AUC are accuracy measures specific to a machine learning model, which correspond to positive predictive value, sensitivity, harmonic mean of precision and recall, and area under the curve, respectively. All measures range from 0 to 1, with a higher value indicating better performance.

**Supple. Table S5 Accuracy of the multiclass XG Boost machine learning models.**

|  | Prediction for the Primary Four Cohort | | |
| --- | --- | --- | --- |
| Prediction Window | All predictors | Top six important predictors ^a^ | Top three important predictors ^b^ |
| Primary Five | 0.85 | 0.78 | 0.74 |
| Primary Six | 0.81 | 0.77 | 0.72 |
| Secondary One | 0.79 | 0.74 | 0.70 |
| Secondary Two | 0.77 | 0.73 | 0.68 |
| Secondary Three | 0.76 | 0.71 | 0.67 |
| Secondary Four | 0.75 | 0.70 | 0.65 |
| Secondary Five | 0.74 | 0.68 | 0.63 |
| Secondary Six | 0.72 | 0.66 | 0.60 |
|  | Prediction for the Primary Six Cohort | | |
| Prediction Window | All predictors | Top six important predictors ^a^ | Top three important predictors ^b^ |
| Secondary One | 0.85 | 0.80 | 0.77 |
| Secondary Two | 0.81 | 0.79 | 0.74 |
| Secondary Three | 0.79 | 0.77 | 0.72 |
| Secondary Four | 0.78 | 0.75 | 0.70 |
| Secondary Five | 0.76 | 0.73 | 0.68 |
| Secondary Six | 0.74 | 0.71 | 0.65 |

XG Boost eXtreme Gradient Boosting

^a^ top six important predictors by the Shapely values were: weight, height, sex, age, hours of aerobic exercise, and frequency of aerobic exercise.

^b^ top three important predictors by the Shapely values were: weight, height, sex.

| **Supple. Table S6** Baseline characteristics of children with gender-specification in two prediction cohorts | | | | |  |
| --- | --- | --- | --- | --- | --- |
| **Characteristics** | | **Primary Four Enrollment Group N_1_ = 442 898** | | **Primary Six Enrollment Group N_2_ = 344 186** | |
|  |  | **Male Prediction Group** | **Female Prediction Group** | **Male Prediction Group** | **Female Prediction Group** |
|  |  | n = 224 398 | n = 218 500 | n = 171 768 | n = 172 418 |
| **Academic grade of follow-up measurement** | | | | |  |
|  | Primary Five | 224 398 | 218 500 | - | - |
|  | Primary Six | 209 394 | 204 796 | - | - |
|  | Secondary One | 181 286 | 181 637 | 171 768 | 172 418 |
|  | Secondary Two | 140 737 | 145 302 | 135 311 | 140 117 |
|  | Secondary Three | 102 390 | 111 428 | 102 116 | 112 288 |
|  | Secondary Four | 76 140 | 86 166 | 80 858 | 92 032 |
|  | Secondary Five | 49 305 | 58 450 | 52 363 | 63 055 |
|  | Secondary Six | 27 183 | 33 091 | 27 998 | 34 505 |
| **Age,** mean (SD), years | | 9.4 (0.55) | 9.4 (0.55) | 11.3 (0.53) | 11.3 (0.53) |
| **Weight,** mean (SD), kg | | 34.3 (8.33) | 32.6 (7.39) | 42.7 (10.54) | 41.4 (8.90) |
| **Height,** mean (SD), cm | | 137.3 (6.48) | 137.6 (7.06) | 149.2 (8.12) | 149.9 (6.89) |
| **Weight Status** | |  | | |  |
|  | Normal | 133 691 (59.6) | 146 577 (67.1) | 111 993 (65.2) | 124 389 (72.1) |
|  | Underweight | 21 854 (9.7) | 30 657 (14.0) | 16 517 (9.6) | 23 725 (13.8) |
|  | Overweight | 51 948 (23.1) | 34 334 (15.7) | 33 928 (19.8) | 20 709 (12.0) |
|  | Obese | 16 905 (7.5) | 6 932 (3.2) | 9 230 (5.4) | 3 695 (2.1) |
| **Breakfast Eating Habit** | | | | |  |
|  | Missing value | 231 (0.1) | 199 (0.1) | 176 (0.1) | 118 (0.1) |
|  | home | 193 484 (86.2) | 192 257 (88.0) | 143 598 (83.7) | 146 921 (85.2) |
|  | rarely at home | 19 468 (8.7) | 15 853 (7.3) | 16 764 (9.8) | 14 334 (8.3) |
|  | no breakfast | 11 215 (5.0) | 10 191 (4.6) | 11 130 (6.5) | 11 145 (6.5) |
| **Sweetness Preference during Past 7 days** | | | | |  |
|  | Missing value | 606 (0.2) | 492 (0.2) | 356 (0.2) | 250 (0.1) |
|  | I like them very much | 71 750 (32.0) | 73 399 (33.6) | 44 674 (26.0) | 59 154 (34.3) |
|  | they are acceptable | 125 676 (56.0) | 127 296 (58.3) | 111 224 (64.8) | 104 953 (60.84) |
|  | I'll try a little | 17 254 (7.7) | 12 291 (5.6) | 10 486 (6.1) | 6 098 (3.5) |
|  | I dislike them | 9 112 (4.1) | 5 022 (2.3) | 4 928 (2.9) | 2 063 (1.2) |
| **Junk Food Intake Habits** | | | | |  |
|  | Missing value | 634 (0.3) | 508 (0.2) | 391 (0.2) | 292 (0.2) |
|  | every day | 10 833 (4.8) | 12 512 (5.7) | 7 264 (4.2) | 12 226 (7.1) |
|  | Occasionally | 128 719 (57.4) | 129 143 (63.7) | 102 737 (59.8) | 117 525 (68.1) |
|  | Rarely | 79 577 (35.5) | 64 175 (29.4) | 58 451 (34.1) | 41 562 (24.1) |
|  | Never | 4 635 (2.1) | 2 162 (1.0) | 2 825 (1.6) | 913 (0.5) |
| **Fruit/ vegetable Intake** | | | | |  |
|  | Missing value | 9 207 (4.1) | 8 983 (4.1) | 8 416 (4.9) | 7 957 (4.6) |
|  | at least thrice a day | 53 829 (24.0) | 57 811 (26.5) | 35 457 (20.7) | 38 633 (22.4) |
|  | once or twice a day | 119 085 (53.1) | 120 754 (55.3) | 95 172 (55.4) | 101 176 (58.7) |
|  | once every few days | 32 226 (14.4) | 24 774 (11.3) | 26 873 (15.7) | 21 438 (12.4) |
|  | less than once a week | 10 051 (4.5) | 6 178 (2.8) | 5 750 (3.4) | 3 314 (1.9) |
| **Milk Consumption Habit** | | | | |  |
|  | Missing value | 843 (0.4) | 616 (0.3) | 493 (0.3) | 302 (0.2) |
|  | at least once a day | 72 213 (32.2) | 67 267 (30.8) | 46 035 (26.8) | 43 429 (25.2) |
|  | once every few days | 62 673 (27.9) | 67 338 (30.8) | 49 979 (29.1) | 53 242 (30.9) |
|  | less than once a week | 43 049 (19.2) | 41 616 (19.0) | 38 946 (22.7) | 40 326 (23.4) |
|  | Never | 45 620 (20.3) | 41 663 (19.1) | 36 215 (21.1) | 35 219 (20.4) |
| **Frequency of Aerobic Exercise** | | | | |  |
|  | Missing value | 3 675 (1.6) | 3 439 (1.6) | 2 456 (1.4) | 2 236 (1.3) |
|  | at least thrice a day | 76 982 (34.3) | 56 317 (25.8) | 59 770 (34.8) | 35 895 (20.8) |
|  | once or twice a day | 93 962 (41.9) | 105 665 (48.4) | 76 039 (44.3) | 86 170 (49.4) |
|  | once every few days | 35 303 (15.7) | 38 127 (17.5) | 25 434 (14.8) | 36 450 (21.1) |
|  | less than once a week | 14 494 (6.5) | 14 952 (6.9) | 7 969 (4.6) | 11 767 (6.8) |
| **Hours of Aerobic Exercise** | | | | |  |
|  | Missing value | 3 674 (1.6) | 3 544 (1.6) | 2 631 (1.5) | 2 416 (1.4) |
|  | more than an hour | 87 823 (39.1) | 54 909 (25.1) | 55 070 (32.1) | 50 004 (29.0) |
|  | half to one hour | 80 495 (35.9) | 94 906 (43.4) | 82 584 (48.1) | 70 089 (40.6) |
|  | less than half an hour | 36 014 (16.1) | 47 701 (21.8) | 22 351 (13.0) | 36 440 (21.1) |
|  | zero | 16 392 (7.3) | 17 440 (8.0) | 9 032 (5.3) | 13 569 (7.9) |
| **Daily Hours of TV Viewing** | | | | |  |
|  | Missing value | 2 928 (1.3) | 1 889 (0.9) | 2 218 (1.3) | 1 590 (0.9) |
|  | less than an hour | 44 172 (19.7) | 45 778 (21.0) | 24 629 (14.3) | 20 393 (11.8) |
|  | one to two hours | 86 380 (38.5) | 89 490 (41.0) | 64 316 (37.5) | 58 509 (33.9) |
|  | two to four hours | 60 539 (27.0) | 59 149 (27.1) | 56 340 (32.8) | 67 178 (38.9) |
|  | more than four hours | 30 379 (13.5) | 21 813 (10.0) | 24 165 (14.1) | 24 848 (14.4) |
| **SEI Score ^a^,** mean (SD) | | | | |  |
|  | Total | 37.4 (7.11) | 38.8 (6.59) | 38.4 (7.11) | 39.3 (6.76) |
|  | General | 15.6 (3.29) | 16.2 (3.17) | 16.2 (3.27) | 16.5 (3.20) |
|  | Social | 7.4 (1.83) | 7.6 (1.79) | 7.9 (1.82) | 7.9 (1.77) |
|  | School-related | 8.0 (1.89) | 8.2 (1.7) | 8.0 (1.93) | 8.2 (1.85) |
|  | Lie (exclude score ≤ 2) | 5.9 (1.72) | 5.8 (1.71) | 6.2 (1.6) | 6.3 (1.7) |
|  | Parent-related | 9.3 (2.01) | 9.8 (1.64) | 9.4 (2.03) | 8.2 (1.85) |
| **RBQ Score ^b^,** mean (SD) | | | | |  |
|  | Total | 11.2 (6.04) | 9.5 (5.42) | 10.2 (5.75) | 8.7 (5.10) |
|  | Conduct | 2.7 (1.37) | 2.3 (1.19) | 2.6 (1.32) | 2.2 (1.13) |
|  | Emotion | 2.4 (1.26) | 2.4 (1.25) | 2.35 (1.24) | 2.5 (1.24) |
|  | Hyperactivity | 3.1 (1.60) | 2.4 (1.43) | 2.7 (1.52) | 2.5 (1.24) |
| **Educational Level of Student's Father** | | | | |  |
|  | Missing value | 9 234 (4.1) | 9 244 (4.2) | 6 512 (3.8) | 6 800 (4.0) |
|  | No Schooling | 1 187 (0.5) | 1 175 (0.5) | 820 (0.5) | 911 (0.5) |
|  | Kindergarten | 216 (0.1) | 234 (0.1) | 145 (0.1) | 168 (0.1) |
|  | Primary | 31 848 (14.2) | 31 030 (14.2) | 24 935 (14.5) | 25 692 (14.9) |
|  | Lower Secondary | 46 705 (20.8) | 45 042 (20.6) | 35 793 (20.9) | 35 808 (20.8) |
|  | Upper Secondary | 88 833 (39.6) | 86 579 (39.6) | 69 020 (40.2) | 69 071 (40.0) |
|  | Matriculation | 8 952 (4.0) | 8 731 (4.0) | 7 074 (4.1) | 7 035 (4.1) |
|  | Tertiary (Non-degree Course) | 9 309 (4.2) | 8 886 (4.1) | 7 372 (4.3) | 7 356 (4.3) |
|  | Tertiary (Degree Course) | 28 114 (12.5) | 27 579 (12.63) | 19 988 (11.6) | 19 677 (11.4) |
| **Educational Level of Student's Mother** | | | | |  |
|  | Missing value | 4 438 (2.0) | 4 217 (1.9) | 3 008 (1.8) | 2 928 (1.7) |
|  | No Schooling | 1 730 (0.8) | 1 802 (0.8) | 1 288 (0.8) | 1 485 (0.9) |
|  | Kindergarten | 222 (0.1) | 224 (0.1) | 171 (0.1) | 194 (0.1) |
|  | Primary | 31 894 (14.2) | 31 198 (14.3) | 26 140 (15.2) | 26 794 (15.5) |
|  | Lower Secondary | 47 677 (21.2) | 46 124 (21.1) | 35 628 (20.8) | 36 025 (20.9) |
|  | Upper Secondary | 103 660 (46.2) | 100 916 (46.2) | 81 103 (47.2) | 80 941 (46.9) |
|  | Matriculation | 8 977 (4.0) | 8 902 (4.1) | 6 790 (4.0) | 6 868 (4.0) |
|  | Tertiary (Non-degree Course) | 7 946 (3.5) | 7 684 (3.5) | 5 914 (3.5) | 5 859 (3.4) |
|  | Tertiary (Degree Course) | 17 854 (8.0) | 17 433 (8.0) | 11 626 (6.8) | 11 424 (6.6) |
| **Occupation of Student's Father** | | | | |  |
|  | Missing value | 9 294 (4.1) | 9 230 (4.2) | 6 438 (3.8) | 6 592 (3.8) |
|  | Managers and Administrators | 24 218 (10.8) | 23 398 (10.7) | 19 305 (11.3) | 19 268 (11.2) |
|  | Professionals | 12 123 (5.4) | 11 942 (5.5) | 9 229 (5.4) | 9 040 (5.2) |
|  | Associate Professionals | 14 395 (6.4) | 13 766 (6.3) | 11 311 (6.6) | 11 308 (6.6) |
|  | Clerks | 21 017 (9.4) | 20 546 (9.4) | 16 610 (9.7) | 16 592 (9.6) |
|  | Service Workers and Shop Sales Workers | 42 306 (18.9) | 40 859 (18.7) | 31 347 (18.3) | 31 465 (18.2) |
|  | Craft and Related Workers | 33 571 (15.0) | 32 652 (14.9) | 27 110 (15.8) | 27 063 (15.7) |
|  | Plant & Machine Operators and Assemblers | 33 168 (14.8) | 32 575 (14.9) | 25 745 (15.0) | 26 304 (15.3) |
|  | Elementary Occupations | 26 415 (11.8) | 25 766 (11.8) | 19 262 (11.2) | 19 487 (11.3) |
|  | Unemployed | 7 891 (3.5) | 7 766 (3.6) | 5 311 (3.1) | 5 399 (3.1) |
| **Occupation of Student's Mother** | | | | |  |
|  | Missing value | 3 200 (1.4) | 2 958 (1.4) | 2 022 (1.2) | 1 909 (1.1) |
|  | Managers and Administrators | 6 593 (2.9) | 6 644 (3.0) | 4 776 (2.8) | 4 950 (2.9) |
|  | Professionals | 4 277 (1.9) | 4 275 (2.0) | 2 988 (1.7) | 3 020 (1.8) |
|  | Associate Professionals | 10 156 (4.5) | 9 972 (4.6) | 7 783 (4.5) | 7 724 (4.5) |
|  | Clerks | 32 279 (16.6) | 37 042 (17.0) | 30 043 (17.5) | 30 309 (17.6) |
|  | Service Workers and Shop Sales Workers | 26 555 (11.8) | 25 976 (11.9) | 17 864 (10.4) | 18 379 (10.7) |
|  | Craft and Related Workers | 2 258 (1.0) | 2 163 (1.0) | 1 776 (1.0) | 1 782 (1.0) |
|  | Plant & Machine Operators and Assemblers | 800 (0.4) | 772 (0.4) | 612 (0.4) | 587 (0.30) |
|  | Elementary Occupations | 9 242 (4.1) | 8 512 (3.9) | 6 496 (3.8) | 6 174 (3.6) |
|  | Unemployed | 124 038 (55.3) | 120 186 (55.0) | 97 308 (56.7) | 97 684 (56.6) |
| **Type of Housing** | | | | |  |
|  | Missing value | 3 753 (1.7) | 3 698 (1.7) | 2 821 (1.7) | 2 879 (1.7) |
|  | Block-Self-contained | 92 607 (41.3) | 90 092 (41.2) | 72 801 (42.4) | 72 558 (42.1) |
|  | Block-non-Self-contained | 5 210 (2.3) | 5 049 (2.3) | 3 243 (1.9) | 3 218 (1.9) |
|  | Housing Authority Home Ownership Estate | 35 779 (15.9) | 34 208 (15.7) | 29 292 (17.1) | 28 702 (16.6) |
|  | Housing Authority/Society Blocks | 76 723 (34.2) | 75 230 (34.4) | 55 858 (32.5) | 57 400 (33.3) |
|  | Village Houses | 8 288 (3.7) | 8 092 (3.7) | 5 880 (3.4) | 5 976 (3.5) |
|  | Institution | 2 128 (1.0) | 2 131 (1.0) | 1 773 (1.0) | 1 794 (1.0) |
| Data are n (%) unless otherwise stated. | | | | | |
| ^a.^ SEI: Culture Free Self-Esteem Inventory for Children Questionnaire. | | | | | |
| ^b.^ RBQ: Rutter Behaviour Questionnaire. | | | | | |

| **Supple. Table S7** Prediction accuracy of different machine learning models with gender-specification. | | | | | | | |
| --- | --- | --- | --- | --- | --- | --- | --- |
| **(a) Male group prediction based on primary four cohort** | | | | | | | |
| **Prediction Window** | **Prediction Performances** | **Random Forest** | **XG Boost** | **Decision Tree** | **k-NN** | **Logistic Regression** | **Supportive Vector Machine** |
| Primary Five | Accuracy | 0.83 | 0.83 | 0.76 | 0.70 | 0.73 | 0.73 |
|  | Micro-AUC | 0.96 | 0.97 | 0.84 | 0.87 | 0.87 | 0.87 |
|  | Macro-AUC | 0.94 | 0.96 | 0.81 | 0.89 | 0.89 | 0.89 |
| Primary Six | Accuracy | 0.78 | 0.79 | 0.71 | 0.64 | 0.69 | 0.70 |
|  | Micro-AUC | 0.94 | 0.95 | 0.81 | 0.84 | 0.86 | 0.88 |
|  | Macro-AUC | 0.92 | 0.93 | 0.77 | 0.85 | 0.87 | 0.89 |
| Secondary One | Accuracy | 0.76 | 0.77 | 0.68 | 0.60 | 0.64 | 0.66 |
|  | Micro-AUC | 0.93 | 0.94 | 0.79 | 0.80 | 0.84 | 0.86 |
|  | Macro-AUC | 0.89 | 0.91 | 0.72 | 0.79 | 0.86 | 0.87 |
| Secondary Two | Accuracy | 0.74 | 0.75 | 0.65 | 0.56 | 0.62 | 0.63 |
|  | Micro-AUC | 0.92 | 0.93 | 0.77 | 0.77 | 0.83 | 0.84 |
|  | Macro-AUC | 0.86 | 0.89 | 0.69 | 0.76 | 0.84 | 0.86 |
| Secondary Three | Accuracy | 0.72 | 0.73 | 0.63 | 0.54 | 0.68 | 0.69 |
|  | Micro-AUC | 0.91 | 0.92 | 0.76 | 0.76 | 0.81 | 0.83 |
|  | Macro-AUC | 0.84 | 0.87 | 0.66 | 0.74 | 0.83 | 0.84 |
| Secondary Four | Accuracy | 0.72 | 0.73 | 0.62 | 0.52 | 0.57 | 0.58 |
|  | Micro-AUC | 0.91 | 0.92 | 0.75 | 0.74 | 0.80 | 0.82 |
|  | Macro-AUC | 0.82 | 0.85 | 0.65 | 0.72 | 0.82 | 0.83 |
| Secondary Five | Accuracy | 0.69 | 0.72 | 0.60 | 0.50 | 0.54 | 0.56 |
|  | Micro-AUC | 0.90 | 0.91 | 0.74 | 0.73 | 0.79 | 0.81 |
|  | Macro-AUC | 0.80 | 0.84 | 0.63 | 0.70 | 0.80 | 0.81 |
| Secondary Six | Accuracy | 0.68 | 0.69 | 0.58 | 0.48 | 0.53 | 0.54 |
|  | Micro-AUC | 0.89 | 0.90 | 0.73 | 0.72 | 0.79 | 0.80 |
|  | Macro-AUC | 0.77 | 0.82 | 0.63 | 0.68 | 0.77 | 0.79 |
|  |  |  |  |  |  |  |  |
| **(b) Male group prediction based on primary six cohort** | | | | | | | |
| **Prediction Window** | **Prediction Performances** | **Random Forest** | **XG Boost** | **Decision Tree** | **k-NN** | **Logistic Regression** | **Supportive Vector Machine** |
| Secondary One | Accuracy | 0.83 | 0.84 | 0.77 | 0.71 | 0.75 | 0.77 |
|  | Micro-AUC | 0.96 | 0.97 | 0.85 | 0.87 | 0.87 | 0.89 |
|  | Macro-AUC | 0.94 | 0.95 | 0.80 | 0.87 | 0.88 | 0.90 |
| Secondary Two | Accuracy | 0.78 | 0.79 | 0.71 | 0.64 | 0.67 | 0.68 |
|  | Micro-AUC | 0.94 | 0.95 | 0.81 | 0.82 | 0.85 | 0.87 |
|  | Macro-AUC | 0.90 | 0.92 | 0.73 | 0.81 | 0.87 | 0.88 |
| Secondary Three | Accuracy | 0.76 | 0.77 | 0.68 | 0.60 | 0.64 | 0.65 |
|  | Micro-AUC | 0.93 | 0.94 | 0.79 | 0.80 | 0.84 | 0.85 |
|  | Macro-AUC | 0.87 | 0.90 | 0.70 | 0.78 | 0.85 | 0.86 |
| Secondary Four | Accuracy | 0.74 | 0.75 | 0.66 | 0.56 | 0.60 | 0.62 |
|  | Micro-AUC | 0.92 | 0.93 | 0.77 | 0.77 | 0.83 | 0.84 |
|  | Macro-AUC | 0.85 | 0.88 | 0.67 | 0.75 | 0.83 | 0.85 |
| Secondary Five | Accuracy | 0.72 | 0.74 | 0.64 | 0.55 | 0.59 | 0.61 |
|  | Micro-AUC | 0.91 | 0.93 | 0.76 | 0.77 | 0.82 | 0.83 |
|  | Macro-AUC | 0.83 | 0.87 | 0.65 | 0.74 | 0.82 | 0.83 |
| Secondary Six | Accuracy | 0.71 | 0.71 | 0.62 | 0.53 | 0.58 | 0.60 |
|  | Micro-AUC | 0.90 | 0.92 | 0.75 | 0.75 | 0.81 | 0.82 |
|  | Macro-AUC | 0.81 | 0.85 | 0.72 | 0.63 | 0.80 | 0.81 |
| **(c) Female group prediction based on primary four cohort** | | | | | | | |
| **Prediction Window** | **Prediction Performances** | **Random Forest** | **XG Boost** | **Decision Tree** | **k-NN** | **Logistic Regression** | **Supportive Vector Machine** |
| Primary Five | Accuracy | 0.84 | 0.85 | 0.78 | 0.72 | 0.75 | 0.75 |
|  | Micro-AUC | 0.96 | 0.97 | 0.86 | 0.88 | 0.88 | 0.88 |
|  | Macro-AUC | 0.94 | 0.96 | 0.81 | 0.89 | 0.86 | 0.86 |
| Primary Six | Accuracy | 0.80 | 0.82 | 0.73 | 0.66 | 0.70 | 0.72 |
|  | Micro-AUC | 0.95 | 0.96 | 0.82 | 0.84 | 0.84 | 0.86 |
|  | Macro-AUC | 0.91 | 0.94 | 0.76 | 0.85 | 0.85 | 0.87 |
| Secondary One | Accuracy | 0.78 | 0.80 | 0.71 | 0.61 | 0.66 | 0.67 |
|  | Micro-AUC | 0.94 | 0.95 | 0.81 | 0.81 | 0.84 | 0.86 |
|  | Macro-AUC | 0.89 | 0.91 | 0.71 | 0.79 | 0.84 | 0.85 |
| Secondary Two | Accuracy | 0.78 | 0.79 | 0.70 | 0.58 | 0.61 | 0.63 |
|  | Micro-AUC | 0.94 | 0.95 | 0.80 | 0.79 | 0.83 | 0.84 |
|  | Macro-AUC | 0.86 | 0.89 | 0.68 | 0.75 | 0.83 | 0.84 |
| Secondary Three | Accuracy | 0.78 | 0.79 | 0.70 | 0.56 | 0.58 | 0.60 |
|  | Micro-AUC | 0.94 | 0.95 | 0.79 | 0.78 | 0.82 | 0.83 |
|  | Macro-AUC | 0.83 | 0.87 | 0.65 | 0.73 | 0.81 | 0.82 |
| Secondary Four | Accuracy | 0.76 | 0.78 | 0.67 | 0.55 | 0.57 | 0.58 |
|  | Micro-AUC | 0.93 | 0.94 | 0.79 | 0.77 | 0.81 | 0.82 |
|  | Macro-AUC | 0.82 | 0.86 | 0.63 | 0.72 | 0.79 | 0.80 |
| Secondary Five | Accuracy | 0.74 | 0.76 | 0.64 | 0.52 | 0.54 | 0.55 |
|  | Micro-AUC | 0.92 | 0.93 | 0.77 | 0.75 | 0.80 | 0.81 |
|  | Macro-AUC | 0.79 | 0.84 | 0.61 | 0.70 | 0.77 | 0.78 |
| Secondary Six | Accuracy | 0.72 | 0.74 | 0.61 | 0.53 | 0.56 | 0.57 |
|  | Micro-AUC | 0.91 | 0.92 | 0.75 | 0.75 | 0.80 | 0.81 |
|  | Macro-AUC | 0.79 | 0.83 | 0.62 | 0.69 | 0.74 | 0.76 |
|  |  |  |  |  |  |  |  |
| **(d) Female group prediction based on primary six cohort** | | | | | | | |
| **Prediction Window** | **Prediction Performances** | **Random Forest** | **XG Boost** | **Decision Tree** | **k-NN** | **Logistic Regression** | **Supportive Vector Machine** |
| Secondary One | Accuracy | 0.85 | 0.86 | 0.79 | 0.73 | 0.74 | 0.76 |
|  | Micro-AUC | 0.97 | 0.98 | 0.86 | 0.88 | 0.88 | 0.89 |
|  | Macro-AUC | 0.94 | 0.96 | 0.79 | 0.87 | 0.86 | 0.87 |
| Secondary Two | Accuracy | 0.83 | 0.83 | 0.76 | 0.68 | 0.69 | 0.71 |
|  | Micro-AUC | 0.96 | 0.97 | 0.84 | 0.85 | 0.86 | 0.87 |
|  | Macro-AUC | 0.91 | 0.93 | 0.74 | 0.82 | 0.84 | 0.86 |
| Secondary Three | Accuracy | 0.81 | 0.82 | 0.73 | 0.64 | 0.65 | 0.66 |
|  | Micro-AUC | 0.95 | 0.96 | 0.82 | 0.83 | 0.84 | 0.85 |
|  | Macro-AUC | 0.88 | 0.91 | 0.70 | 0.79 | 0.83 | 0.84 |
| Secondary Four | Accuracy | 0.78 | 0.79 | 0.70 | 0.61 | 0.63 | 0.64 |
|  | Micro-AUC | 0.94 | 0.95 | 0.81 | 0.81 | 0.83 | 0.85 |
|  | Macro-AUC | 0.85 | 0.89 | 0.67 | 0.76 | 0.81 | 0.82 |
| Secondary Five | Accuracy | 0.76 | 0.77 | 0.68 | 0.58 | 0.59 | 0.61 |
|  | Micro-AUC | 0.93 | 0.94 | 0.78 | 0.79 | 0.82 | 0.83 |
|  | Macro-AUC | 0.84 | 0.87 | 0.75 | 0.74 | 0.79 | 0.80 |
| Secondary Six | Accuracy | 0.73 | 0.75 | 0.65 | 0.57 | 0.61 | 0.62 |
|  | Micro-AUC | 0.92 | 0.93 | 0.79 | 0.77 | 0.82 | 0.83 |
|  | Macro-AUC | 0.82 | 0.87 | 0.71 | 0.64 | 0.77 | 0.78 |
| XG Boost eXtreme Gradient Boosting; AUC area under the curve. | | | | | | | |

| **Supple. Table S8** AUC values of prediction models by weight status. | | | | | | | | | |
| --- | --- | --- | --- | --- | --- | --- | --- | --- | --- |
| **(a) Male group prediction based on the primary four cohort** | | | | | | | | | |
| **Weight Status** | **Machine Learning Model** | **Primary Five** | **Primary Six** | **Secondary One** | **Secondary Two** | **Secondary Three** | **Secondary Four** | **Secondary Five** | **Secondary Six** |
| Normal | Random Forest | 0.924 | 0.882 | 0.845 | 0.801 | 0.762 | 0.734 | 0.702 | 0.668 |
|  | XG Boost | 0.936 | 0.898 | 0.864 | 0.821 | 0.786 | 0.759 | 0.735 | 0.698 |
|  | Decision Tree | 0.807 | 0.754 | 0.713 | 0.675 | 0.644 | 0.619 | 0.601 | 0.568 |
|  | k-Nearest Neighbors | 0.844 | 0.798 | 0.753 | 0.712 | 0.683 | 0.658 | 0.641 | 0.594 |
|  | Logistic Regression | 0.744 | 0.626 | 0.609 | 0.582 | 0.539 | 0.498 | 0.459 | 0.422 |
|  | Support Vector machine | 0.889 | 0.771 | 0.754 | 0.727 | 0.689 | 0.648 | 0.609 | 0.572 |
| Underweight | Random Forest | 0.952 | 0.936 | 0.911 | 0.884 | 0.869 | 0.860 | 0.845 | 0.831 |
|  | XG Boost | 0.970 | 0.956 | 0.941 | 0.917 | 0.901 | 0.894 | 0.872 | 0.855 |
|  | Decision Tree | 0.808 | 0.775 | 0.736 | 0.702 | 0.687 | 0.680 | 0.669 | 0.675 |
|  | k-Nearest Neighbors | 0.911 | 0.892 | 0.851 | 0.816 | 0.800 | 0.794 | 0.786 | 0.754 |
|  | Logistic Regression | 0.811 | 0.847 | 0.834 | 0.813 | 0.802 | 0.803 | 0.780 | 0.765 |
|  | Support Vector machine | 0.861 | 0.897 | 0.884 | 0.863 | 0.852 | 0.853 | 0.830 | 0.815 |
| Overweight | Random Forest | 0.926 | 0.889 | 0.861 | 0.836 | 0.824 | 0.798 | 0.801 | 0.766 |
|  | XG Boost | 0.942 | 0.908 | 0.879 | 0.862 | 0.845 | 0.834 | 0.829 | 0.816 |
|  | Decision Tree | 0.785 | 0.723 | 0.680 | 0.655 | 0.639 | 0.624 | 0.609 | 0.619 |
|  | k-Nearest Neighbors | 0.861 | 0.811 | 0.751 | 0.727 | 0.709 | 0.689 | 0.690 | 0.686 |
|  | Logistic Regression | 0.761 | 0.735 | 0.736 | 0.726 | 0.725 | 0.719 | 0.726 | 0.701 |
|  | Support Vector machine | 0.818 | 0.792 | 0.793 | 0.785 | 0.784 | 0.778 | 0.786 | 0.761 |
| Obese | Random Forest | 0.968 | 0.954 | 0.935 | 0.914 | 0.907 | 0.875 | 0.836 | 0.816 |
|  | XG Boost | 0.985 | 0.976 | 0.964 | 0.950 | 0.940 | 0.930 | 0.907 | 0.891 |
|  | Decision Tree | 0.848 | 0.809 | 0.752 | 0.709 | 0.679 | 0.674 | 0.633 | 0.644 |
|  | k-Nearest Neighbors | 0.926 | 0.912 | 0.821 | 0.779 | 0.753 | 0.733 | 0.693 | 0.674 |
|  | Logistic Regression | 0.826 | 0.870 | 0.860 | 0.850 | 0.846 | 0.838 | 0.818 | 0.801 |
|  | Support Vector machine | 0.883 | 0.927 | 0.917 | 0.910 | 0.906 | 0.898 | 0.868 | 0.851 |
| **(b) Male group prediction based on the primary six cohort** | | | | | | | | | |
| **Weight Status** | **Machine Learning Model** | **Secondary One** | **Secondary Two** | **Secondary Three** | **Secondary Four** | **Secondary Five** | **Secondary Six** |  |  |
| Normal | Random Forest | 0.912 | 0.855 | 0.815 | 0.773 | 0.752 | 0.725 |  |  |
|  | XG Boost | 0.927 | 0.874 | 0.838 | 0.792 | 0.776 | 0.747 |  |  |
|  | Decision Tree | 0.792 | 0.722 | 0.685 | 0.644 | 0.633 | 0.618 |  |  |
|  | k-Nearest Neighbors | 0.838 | 0.778 | 0.736 | 0.699 | 0.686 | 0.659 |  |  |
|  | Logistic Regression | 0.627 | 0.598 | 0.557 | 0.522 | 0.493 | 0.441 |  |  |
|  | Support Vector machine | 0.772 | 0.743 | 0.707 | 0.672 | 0.643 | 0.591 |  |  |
| Underweight | Random Forest | 0.925 | 0.884 | 0.854 | 0.837 | 0.826 | 0.811 |  |  |
|  | XG Boost | 0.940 | 0.903 | 0.883 | 0.864 | 0.855 | 0.844 |  |  |
|  | Decision Tree | 0.767 | 0.711 | 0.673 | 0.648 | 0.633 | 0.607 |  |  |
|  | k-Nearest Neighbors | 0.845 | 0.787 | 0.749 | 0.729 | 0.714 | 0.703 |  |  |
|  | Logistic Regression | 0.764 | 0.760 | 0.749 | 0.748 | 0.743 | 0.730 |  |  |
|  | Support Vector machine | 0.814 | 0.810 | 0.799 | 0.798 | 0.793 | 0.780 |  |  |
| Overweight | Random Forest | 0.943 | 0.912 | 0.889 | 0.878 | 0.868 | 0.857 |  |  |
|  | XG Boost | 0.964 | 0.939 | 0.919 | 0.907 | 0.897 | 0.885 |  |  |
|  | Decision Tree | 0.793 | 0.747 | 0.715 | 0.706 | 0.700 | 0.696 |  |  |
|  | k-Nearest Neighbors | 0.898 | 0.853 | 0.833 | 0.814 | 0.824 | 0.802 |  |  |
|  | Logistic Regression | 0.857 | 0.835 | 0.822 | 0.811 | 0.801 | 0.791 |  |  |
|  | Support Vector machine | 0.914 | 0.894 | 0.881 | 0.870 | 0.861 | 0.851 |  |  |
| Obese | Random Forest | 0.969 | 0.937 | 0.923 | 0.891 | 0.884 | 0.853 |  |  |
|  | XG Boost | 0.985 | 0.969 | 0.961 | 0.947 | 0.938 | 0.923 |  |  |
|  | Decision Tree | 0.833 | 0.739 | 0.714 | 0.675 | 0.649 | 0.607 |  |  |
|  | k-Nearest Neighbors | 0.884 | 0.820 | 0.804 | 0.770 | 0.732 | 0.723 |  |  |
|  | Logistic Regression | 0.882 | 0.871 | 0.865 | 0.853 | 0.846 | 0.840 |  |  |
|  | Support Vector machine | 0.939 | 0.931 | 0.925 | 0.913 | 0.896 | 0.890 |  |  |
| **(c) Female group prediction based on the primary four cohort** | | | | | | | | | |
| **Weight Status** | **Machine Learning Model** | **Primary Five** | **Primary Six** | **Secondary One** | **Secondary Two** | **Secondary Three** | **Secondary Four** | **Secondary Five** | **Secondary Six** |
| Normal | Random Forest | 0.913 | 0.869 | 0.820 | 0.777 | 0.748 | 0.720 | 0.699 | 0.699 |
|  | XG Boost | 0.929 | 0.887 | 0.841 | 0.799 | 0.771 | 0.748 | 0.727 | 0.723 |
|  | Decision Tree | 0.791 | 0.739 | 0.687 | 0.649 | 0.617 | 0.607 | 0.608 | 0.597 |
|  | k-Nearest Neighbors | 0.839 | 0.793 | 0.732 | 0.698 | 0.671 | 0.653 | 0.623 | 0.622 |
|  | Logistic Regression | 0.510 | 0.693 | 0.518 | 0.490 | 0.452 | 0.394 | 0.356 | 0.303 |
|  | Support Vector machine | 0.655 | 0.838 | 0.663 | 0.635 | 0.602 | 0.544 | 0.506 | 0.453 |
| Underweight | Random Forest | 0.948 | 0.927 | 0.894 | 0.869 | 0.850 | 0.836 | 0.816 | 0.815 |
|  | XG Boost | 0.964 | 0.947 | 0.924 | 0.904 | 0.880 | 0.863 | 0.843 | 0.839 |
|  | Decision Tree | 0.812 | 0.766 | 0.719 | 0.701 | 0.669 | 0.663 | 0.651 | 0.660 |
|  | k-Nearest Neighbors | 0.901 | 0.879 | 0.833 | 0.809 | 0.783 | 0.763 | 0.748 | 0.748 |
|  | Logistic Regression | 0.851 | 0.779 | 0.822 | 0.804 | 0.784 | 0.763 | 0.744 | 0.744 |
|  | Support Vector machine | 0.901 | 0.829 | 0.872 | 0.854 | 0.834 | 0.813 | 0.794 | 0.794 |
| Overweight | Random Forest | 0.941 | 0.908 | 0.885 | 0.860 | 0.847 | 0.832 | 0.821 | 0.822 |
|  | XG Boost | 0.958 | 0.931 | 0.906 | 0.886 | 0.879 | 0.874 | 0.855 | 0.856 |
|  | Decision Tree | 0.790 | 0.738 | 0.688 | 0.664 | 0.644 | 0.628 | 0.595 | 0.618 |
|  | k-Nearest Neighbors | 0.877 | 0.831 | 0.775 | 0.753 | 0.732 | 0.728 | 0.711 | 0.707 |
|  | Logistic Regression | 0.780 | 0.731 | 0.735 | 0.745 | 0.731 | 0.732 | 0.727 | 0.691 |
|  | Support Vector machine | 0.837 | 0.788 | 0.792 | 0.804 | 0.790 | 0.791 | 0.787 | 0.751 |
| Obese | Random Forest | 0.973 | 0.949 | 0.941 | 0.915 | 0.889 | 0.899 | 0.833 | 0.812 |
|  | XG Boost | 0.989 | 0.984 | 0.975 | 0.971 | 0.963 | 0.960 | 0.952 | 0.917 |
|  | Decision Tree | 0.851 | 0.791 | 0.744 | 0.702 | 0.660 | 0.639 | 0.584 | 0.584 |
|  | k-Nearest Neighbors | 0.923 | 0.902 | 0.799 | 0.753 | 0.746 | 0.723 | 0.715 | 0.662 |
|  | Logistic Regression | 0.885 | 0.802 | 0.874 | 0.873 | 0.864 | 0.870 | 0.862 | 0.838 |
|  | Support Vector machine | 0.942 | 0.859 | 0.931 | 0.933 | 0.924 | 0.930 | 0.912 | 0.888 |
| **(d) Female group prediction based on the primary six cohort** | | | | | | | | | |
| **Weight Status** | **Machine Learning Model** | **Secondary One** | **Secondary Two** | **Secondary Three** | **Secondary Four** | **Secondary Five** | **Secondary Six** |  |  |
| Normal | Random Forest | 0.907 | 0.852 | 0.811 | 0.771 | 0.734 | 0.718 |  |  |
|  | XG Boost | 0.923 | 0.877 | 0.838 | 0.799 | 0.767 | 0.752 |  |  |
|  | Decision Tree | 0.778 | 0.718 | 0.675 | 0.645 | 0.616 | 0.613 |  |  |
|  | k-Nearest Neighbors | 0.836 | 0.783 | 0.747 | 0.704 | 0.673 | 0.661 |  |  |
|  | Logistic Regression | 0.530 | 0.494 | 0.445 | 0.401 | 0.347 | 0.309 |  |  |
|  | Support Vector machine | 0.675 | 0.639 | 0.595 | 0.551 | 0.497 | 0.459 |  |  |
| Underweight | Random Forest | 0.941 | 0.912 | 0.892 | 0.878 | 0.856 | 0.854 |  |  |
|  | XG Boost | 0.956 | 0.935 | 0.921 | 0.907 | 0.893 | 0.896 |  |  |
|  | Decision Tree | 0.781 | 0.730 | 0.691 | 0.673 | 0.639 | 0.640 |  |  |
|  | k-Nearest Neighbors | 0.867 | 0.825 | 0.797 | 0.780 | 0.749 | 0.744 |  |  |
|  | Logistic Regression | 0.766 | 0.770 | 0.770 | 0.781 | 0.766 | 0.731 |  |  |
|  | Support Vector machine | 0.816 | 0.820 | 0.820 | 0.831 | 0.816 | 0.781 |  |  |
| Overweight | Random Forest | 0.943 | 0.911 | 0.888 | 0.860 | 0.842 | 0.830 |  |  |
|  | XG Boost | 0.961 | 0.935 | 0.912 | 0.882 | 0.865 | 0.849 |  |  |
|  | Decision Tree | 0.795 | 0.748 | 0.704 | 0.690 | 0.683 | 0.669 |  |  |
|  | k-Nearest Neighbors | 0.896 | 0.855 | 0.832 | 0.797 | 0.782 | 0.770 |  |  |
|  | Logistic Regression | 0.850 | 0.827 | 0.808 | 0.781 | 0.761 | 0.757 |  |  |
|  | Support Vector machine | 0.907 | 0.886 | 0.867 | 0.840 | 0.821 | 0.817 |  |  |
| Obese | Random Forest | 0.969 | 0.946 | 0.921 | 0.906 | 0.911 | 0.892 |  |  |
|  | XG Boost | 0.991 | 0.982 | 0.977 | 0.976 | 0.968 | 0.963 |  |  |
|  | Decision Tree | 0.799 | 0.748 | 0.733 | 0.682 | 0.650 | 0.638 |  |  |
|  | k-Nearest Neighbors | 0.862 | 0.808 | 0.784 | 0.759 | 0.755 | 0.683 |  |  |
|  | Logistic Regression | 0.889 | 0.885 | 0.878 | 0.882 | 0.871 | 0.870 |  |  |
|  | Support Vector machine | 0.946 | 0.945 | 0.938 | 0.942 | 0.921 | 0.920 |  |  |

AUROC area under the characteristic curve, XG Boost eXtreme Gradient Boosting

| **Supple. Table S9** Prediction performances of each weight status with gender-specification. | | | | | | | | | | | | | | | | | | | | | | |
| --- | --- | --- | --- | --- | --- | --- | --- | --- | --- | --- | --- | --- | --- | --- | --- | --- | --- | --- | --- | --- | --- | --- |
| 1. **Male group prediction** | | | | | | | | | | | | | | | | | | | | | | |
| Prediction Model | | Random Forest | | | | | XG Boost | | | Decision Tree | | | k-Nearest Neighbors | | | Logistic Regression | | | Support Vector Machine | | | |
| Prediction Performance | | Precision | Recall | | F1-score | | Precision | Recall | F1-score | Precision | Recall | F1-score | Precision | Recall | F1-score | Precision | Recall | F1-score | Precision | Recall | F1-score | |
|  |  | Primary Four Prediction Cohort | | | | | | | | | | | | | | | | | | | | |
| Primary Five | Normal | 0.875 | 0.898 | | 0.886 | | 0.894 | 0.884 | 0.889 | 0.858 | 0.815 | 0.836 | 0.902 | 0.656 | 0.760 | 0.902 | 0.715 | 0.798 | 0.835 | 0.858 | 0.846 | |
|  | Underweight | 0.724 | 0.661 | | 0.691 | | 0.721 | 0.739 | 0.730 | 0.568 | 0.680 | 0.618 | 0.403 | 0.855 | 0.547 | 0.455 | 0.869 | 0.597 | 0.684 | 0.621 | 0.651 | |
|  | Overweight | 0.759 | 0.740 | | 0.750 | | 0.754 | 0.763 | 0.758 | 0.661 | 0.666 | 0.664 | 0.635 | 0.728 | 0.678 | 0.663 | 0.709 | 0.685 | 0.719 | 0.700 | 0.710 | |
|  | Obese | 0.759 | 0.740 | | 0.750 | | 0.763 | 0.779 | 0.771 | 0.651 | 0.734 | 0.690 | 0.625 | 0.801 | 0.702 | 0.630 | 0.803 | 0.706 | 0.719 | 0.700 | 0.710 | |
| Primary Six | Normal | 0.843 | 0.875 | | 0.859 | | 0.862 | 0.865 | 0.864 | 0.824 | 0.779 | 0.801 | 0.870 | 0.597 | 0.708 | 0.883 | 0.671 | 0.762 | 0.803 | 0.835 | 0.819 | |
|  | Underweight | 0.677 | 0.589 | | 0.630 | | 0.684 | 0.654 | 0.669 | 0.514 | 0.603 | 0.555 | 0.367 | 0.824 | 0.508 | 0.424 | 0.861 | 0.568 | 0.637 | 0.549 | 0.590 | |
|  | Overweight | 0.672 | 0.641 | | 0.656 | | 0.681 | 0.679 | 0.680 | 0.567 | 0.580 | 0.574 | 0.543 | 0.647 | 0.590 | 0.584 | 0.631 | 0.607 | 0.632 | 0.601 | 0.616 | |
|  | Obese | 0.672 | 0.668 | | 0.670 | | 0.688 | 0.714 | 0.701 | 0.552 | 0.649 | 0.597 | 0.521 | 0.767 | 0.621 | 0.531 | 0.784 | 0.633 | 0.632 | 0.628 | 0.630 | |
| Secondary One | Normal | 0.829 | 0.874 | | 0.851 | | 0.841 | 0.871 | 0.856 | 0.810 | 0.763 | 0.786 | 0.851 | 0.581 | 0.691 | 0.883 | 0.623 | 0.730 | 0.789 | 0.834 | 0.811 | |
|  | Underweight | 0.642 | 0.496 | | 0.560 | | 0.655 | 0.545 | 0.595 | 0.453 | 0.538 | 0.492 | 0.340 | 0.732 | 0.465 | 0.369 | 0.866 | 0.517 | 0.602 | 0.456 | 0.520 | |
|  | Overweight | 0.596 | 0.568 | | 0.582 | | 0.608 | 0.587 | 0.597 | 0.484 | 0.505 | 0.494 | 0.445 | 0.594 | 0.509 | 0.511 | 0.584 | 0.545 | 0.556 | 0.528 | 0.542 | |
|  | Obese | 0.593 | 0.545 | | 0.568 | | 0.601 | 0.600 | 0.601 | 0.457 | 0.550 | 0.500 | 0.407 | 0.618 | 0.490 | 0.460 | 0.749 | 0.570 | 0.553 | 0.505 | 0.528 | |
| Secondary Two | Normal | 0.813 | 0.872 | | 0.841 | | 0.821 | 0.873 | 0.846 | 0.801 | 0.749 | 0.774 | 0.837 | 0.549 | 0.663 | 0.878 | 0.594 | 0.709 | 0.773 | 0.832 | 0.801 | |
|  | Underweight | 0.588 | 0.411 | | 0.484 | | 0.603 | 0.445 | 0.512 | 0.403 | 0.477 | 0.437 | 0.307 | 0.691 | 0.425 | 0.342 | 0.843 | 0.487 | 0.548 | 0.371 | 0.444 | |
|  | Overweight | 0.532 | 0.496 | | 0.513 | | 0.546 | 0.509 | 0.527 | 0.418 | 0.449 | 0.433 | 0.385 | 0.557 | 0.455 | 0.451 | 0.547 | 0.495 | 0.492 | 0.456 | 0.473 | |
|  | Obese | 0.544 | 0.462 | | 0.499 | | 0.553 | 0.498 | 0.524 | 0.382 | 0.472 | 0.422 | 0.335 | 0.525 | 0.409 | 0.412 | 0.700 | 0.518 | 0.504 | 0.422 | 0.459 | |
| Secondary Three | Normal | 0.792 | 0.864 | | 0.826 | | 0.795 | 0.869 | 0.830 | 0.778 | 0.729 | 0.753 | 0.818 | 0.517 | 0.634 | 0.865 | 0.543 | 0.667 | 0.752 | 0.824 | 0.786 | |
|  | Underweight | 0.594 | 0.392 | | 0.472 | | 0.611 | 0.438 | 0.511 | 0.405 | 0.463 | 0.432 | 0.328 | 0.677 | 0.442 | 0.354 | 0.842 | 0.499 | 0.554 | 0.352 | 0.432 | |
|  | Overweight | 0.490 | 0.443 | | 0.465 | | 0.502 | 0.421 | 0.458 | 0.388 | 0.418 | 0.403 | 0.351 | 0.528 | 0.422 | 0.402 | 0.500 | 0.446 | 0.450 | 0.403 | 0.425 | |
|  | Obese | 0.439 | 0.381 | | 0.408 | | 0.490 | 0.461 | 0.475 | 0.317 | 0.417 | 0.360 | 0.253 | 0.479 | 0.331 | 0.336 | 0.715 | 0.457 | 0.399 | 0.341 | 0.368 | |
| Secondary Four | Normal | 0.792 | 0.862 | | 0.825 | | 0.791 | 0.879 | 0.833 | 0.778 | 0.720 | 0.748 | 0.813 | 0.493 | 0.614 | 0.864 | 0.531 | 0.658 | 0.752 | 0.822 | 0.785 | |
|  | Underweight | 0.609 | 0.405 | | 0.487 | | 0.625 | 0.445 | 0.520 | 0.416 | 0.471 | 0.442 | 0.341 | 0.683 | 0.454 | 0.371 | 0.835 | 0.514 | 0.569 | 0.365 | 0.447 | |
|  | Overweight | 0.447 | 0.399 | | 0.421 | | 0.472 | 0.355 | 0.405 | 0.342 | 0.381 | 0.360 | 0.302 | 0.499 | 0.376 | 0.347 | 0.470 | 0.400 | 0.407 | 0.359 | 0.381 | |
|  | Obese | 0.337 | 0.314 | | 0.325 | | 0.392 | 0.358 | 0.374 | 0.242 | 0.362 | 0.290 | 0.197 | 0.458 | 0.276 | 0.269 | 0.688 | 0.387 | 0.297 | 0.274 | 0.285 | |
| Secondary Five | Normal | 0.763 | 0.853 | | 0.805 | | 0.771 | 0.884 | 0.824 | 0.756 | 0.701 | 0.727 | 0.793 | 0.471 | 0.591 | 0.853 | 0.495 | 0.626 | 0.723 | 0.813 | 0.765 | |
|  | Underweight | 0.571 | 0.379 | | 0.455 | | 0.646 | 0.461 | 0.538 | 0.424 | 0.465 | 0.444 | 0.364 | 0.678 | 0.474 | 0.386 | 0.821 | 0.526 | 0.531 | 0.339 | 0.415 | |
|  | Overweight | 0.422 | 0.367 | | 0.393 | | 0.438 | 0.279 | 0.341 | 0.309 | 0.360 | 0.332 | 0.280 | 0.490 | 0.356 | 0.325 | 0.453 | 0.379 | 0.382 | 0.327 | 0.353 | |
|  | Obese | 0.338 | 0.220 | | 0.266 | | 0.379 | 0.296 | 0.332 | 0.199 | 0.268 | 0.229 | 0.173 | 0.393 | 0.240 | 0.234 | 0.652 | 0.344 | 0.298 | 0.180 | 0.226 | |
| Secondary Six | Normal | 0.745 | 0.836 | | 0.788 | | 0.749 | 0.854 | 0.798 | 0.733 | 0.677 | 0.704 | 0.764 | 0.445 | 0.562 | 0.829 | 0.478 | 0.606 | 0.705 | 0.796 | 0.748 | |
|  | Underweight | 0.574 | 0.440 | | 0.498 | | 0.577 | 0.473 | 0.520 | 0.417 | 0.465 | 0.440 | 0.358 | 0.668 | 0.466 | 0.399 | 0.812 | 0.535 | 0.534 | 0.400 | 0.458 | |
|  | Overweight | 0.400 | 0.295 | | 0.339 | | 0.407 | 0.221 | 0.287 | 0.266 | 0.293 | 0.279 | 0.286 | 0.471 | 0.356 | 0.315 | 0.409 | 0.356 | 0.360 | 0.255 | 0.299 | |
|  | Obese | 0.241 | 0.155 | | 0.188 | | 0.218 | 0.179 | 0.196 | 0.131 | 0.236 | 0.169 | 0.111 | 0.342 | 0.167 | 0.149 | 0.602 | 0.239 | 0.201 | 0.115 | 0.148 | |
|  |  | Primary Six Prediction Cohort | | | | | | | | | | | | | | | | | | | | |
| Secondary One | Normal | 0.883 | 0.903 | | 0.893 | | 0.899 | 0.895 | 0.897 | 0.866 | 0.834 | 0.850 | 0.914 | 0.693 | 0.788 | 0.928 | 0.698 | 0.797 | 0.848 | 0.868 | 0.858 | |
|  | Underweight | 0.710 | 0.702 | | 0.706 | | 0.712 | 0.738 | 0.725 | 0.616 | 0.630 | 0.623 | 0.565 | 0.734 | 0.638 | 0.598 | 0.713 | 0.650 | 0.675 | 0.667 | 0.671 | |
|  | Overweight | 0.729 | 0.648 | | 0.686 | | 0.734 | 0.693 | 0.713 | 0.560 | 0.638 | 0.596 | 0.431 | 0.809 | 0.563 | 0.432 | 0.895 | 0.583 | 0.694 | 0.613 | 0.651 | |
|  | Obese | 0.727 | 0.702 | | 0.714 | | 0.728 | 0.741 | 0.734 | 0.597 | 0.663 | 0.628 | 0.566 | 0.729 | 0.637 | 0.579 | 0.812 | 0.676 | 0.692 | 0.667 | 0.679 | |
| Secondary Two | Normal | 0.849 | 0.889 | | 0.868 | | 0.860 | 0.884 | 0.872 | 0.835 | 0.793 | 0.814 | 0.882 | 0.617 | 0.726 | 0.915 | 0.631 | 0.747 | 0.814 | 0.854 | 0.833 | |
|  | Underweight | 0.601 | 0.590 | | 0.596 | | 0.610 | 0.614 | 0.612 | 0.494 | 0.526 | 0.509 | 0.449 | 0.650 | 0.531 | 0.511 | 0.666 | 0.578 | 0.566 | 0.555 | 0.561 | |
|  | Overweight | 0.669 | 0.534 | | 0.594 | | 0.680 | 0.579 | 0.625 | 0.489 | 0.562 | 0.523 | 0.372 | 0.745 | 0.496 | 0.384 | 0.877 | 0.534 | 0.634 | 0.499 | 0.559 | |
|  | Obese | 0.625 | 0.522 | | 0.569 | | 0.639 | 0.581 | 0.609 | 0.469 | 0.538 | 0.501 | 0.440 | 0.618 | 0.514 | 0.509 | 0.756 | 0.608 | 0.590 | 0.487 | 0.534 | |
| Secondary Three | Normal | 0.827 | 0.880 | | 0.853 | | 0.834 | 0.879 | 0.856 | 0.815 | 0.774 | 0.794 | 0.856 | 0.583 | 0.694 | 0.898 | 0.611 | 0.727 | 0.792 | 0.845 | 0.818 | |
|  | Underweight | 0.544 | 0.508 | | 0.525 | | 0.551 | 0.507 | 0.528 | 0.448 | 0.471 | 0.459 | 0.392 | 0.577 | 0.466 | 0.457 | 0.590 | 0.515 | 0.509 | 0.473 | 0.490 | |
|  | Overweight | 0.654 | 0.492 | | 0.562 | | 0.671 | 0.538 | 0.597 | 0.462 | 0.531 | 0.494 | 0.368 | 0.728 | 0.488 | 0.389 | 0.859 | 0.535 | 0.619 | 0.457 | 0.527 | |
|  | Obese | 0.511 | 0.456 | | 0.482 | | 0.537 | 0.520 | 0.528 | 0.377 | 0.461 | 0.415 | 0.336 | 0.568 | 0.422 | 0.408 | 0.727 | 0.522 | 0.476 | 0.421 | 0.447 | |
| Secondary Four | Normal | 0.807 | 0.870 | | 0.838 | | 0.810 | 0.880 | 0.844 | 0.795 | 0.749 | 0.771 | 0.843 | 0.542 | 0.659 | 0.887 | 0.568 | 0.692 | 0.772 | 0.835 | 0.803 | |
|  | Underweight | 0.484 | 0.434 | | 0.458 | | 0.508 | 0.414 | 0.456 | 0.378 | 0.411 | 0.394 | 0.341 | 0.550 | 0.421 | 0.386 | 0.542 | 0.451 | 0.449 | 0.399 | 0.423 | |
|  | Overweight | 0.626 | 0.453 | | 0.526 | | 0.644 | 0.477 | 0.548 | 0.448 | 0.505 | 0.475 | 0.364 | 0.720 | 0.484 | 0.387 | 0.848 | 0.531 | 0.591 | 0.418 | 0.491 | |
|  | Obese | 0.392 | 0.352 | | 0.371 | | 0.448 | 0.459 | 0.453 | 0.310 | 0.414 | 0.355 | 0.266 | 0.513 | 0.350 | 0.323 | 0.673 | 0.437 | 0.357 | 0.317 | 0.336 | |
| Secondary Five | Normal | 0.796 | 0.862 | | 0.828 | | 0.795 | 0.883 | 0.837 | 0.776 | 0.733 | 0.754 | 0.831 | 0.525 | 0.643 | 0.879 | 0.553 | 0.679 | 0.761 | 0.827 | 0.793 | |
|  | Underweight | 0.435 | 0.410 | | 0.422 | | 0.469 | 0.358 | 0.406 | 0.339 | 0.379 | 0.358 | 0.306 | 0.524 | 0.387 | 0.339 | 0.518 | 0.410 | 0.400 | 0.375 | 0.387 | |
|  | Overweight | 0.646 | 0.471 | | 0.545 | | 0.662 | 0.489 | 0.563 | 0.449 | 0.491 | 0.469 | 0.397 | 0.737 | 0.516 | 0.423 | 0.833 | 0.561 | 0.611 | 0.436 | 0.510 | |
|  | Obese | 0.369 | 0.277 | | 0.317 | | 0.403 | 0.342 | 0.370 | 0.286 | 0.353 | 0.316 | 0.243 | 0.454 | 0.316 | 0.299 | 0.636 | 0.407 | 0.334 | 0.242 | 0.282 | |
| Secondary Six | Normal | 0.781 | 0.852 | | 0.815 | | 0.777 | 0.859 | 0.816 | 0.769 | 0.708 | 0.737 | 0.808 | 0.487 | 0.607 | 0.866 | 0.539 | 0.664 | 0.746 | 0.817 | 0.780 | |
|  | Underweight | 0.440 | 0.377 | | 0.406 | | 0.412 | 0.253 | 0.314 | 0.319 | 0.360 | 0.338 | 0.284 | 0.486 | 0.358 | 0.322 | 0.473 | 0.383 | 0.405 | 0.342 | 0.371 | |
|  | Overweight | 0.615 | 0.476 | | 0.537 | | 0.613 | 0.541 | 0.575 | 0.451 | 0.524 | 0.485 | 0.395 | 0.732 | 0.513 | 0.439 | 0.829 | 0.574 | 0.580 | 0.441 | 0.502 | |
|  | Obese | 0.369 | 0.292 | | 0.326 | | 0.282 | 0.240 | 0.260 | 0.209 | 0.286 | 0.241 | 0.188 | 0.442 | 0.264 | 0.244 | 0.669 | 0.358 | 0.334 | 0.257 | 0.291 | |
| 1. **Female group prediction** | | | | | | | | | | | | | | | | | | | | | |  |
| Prediction Model | | Random Forest | | | | | XG Boost | | | Decision Tree | | | k-Nearest Neighbors | | | Logistic Regression | | | Support Vector Machine | | |  |
| Prediction Performance | | Precision | | Recall | | F1-score | Precision | Recall | F1-score | Precision | Recall | F1-score | Precision | Recall | F1-score | Precision | Recall | F1-score | Precision | Recall | F1-score |  |
|  |  | Primary Four Prediction Cohort | | | | | | | | | | | | | | | | | | | |  |
| Primary Five | Normal | 0.886 | | 0.904 | | 0.895 | 0.900 | 0.901 | 0.900 | 0.870 | 0.837 | 0.854 | 0.919 | 0.679 | 0.781 | 0.924 | 0.723 | 0.811 | 0.846 | 0.864 | 0.855 |  |
|  | Underweight | 0.752 | | 0.698 | | 0.724 | 0.756 | 0.744 | 0.750 | 0.632 | 0.691 | 0.660 | 0.479 | 0.849 | 0.612 | 0.526 | 0.878 | 0.658 | 0.712 | 0.658 | 0.684 |  |
|  | Overweight | 0.739 | | 0.718 | | 0.728 | 0.748 | 0.739 | 0.744 | 0.629 | 0.647 | 0.638 | 0.570 | 0.755 | 0.649 | 0.587 | 0.712 | 0.643 | 0.699 | 0.678 | 0.688 |  |
|  | Obese | 0.710 | | 0.733 | | 0.722 | 0.706 | 0.779 | 0.741 | 0.581 | 0.719 | 0.643 | 0.550 | 0.795 | 0.650 | 0.520 | 0.818 | 0.636 | 0.670 | 0.693 | 0.682 |  |
| Primary Six | Normal | 0.862 | | 0.892 | | 0.877 | 0.876 | 0.891 | 0.883 | 0.845 | 0.801 | 0.822 | 0.900 | 0.619 | 0.733 | 0.917 | 0.680 | 0.781 | 0.822 | 0.852 | 0.837 |  |
|  | Underweight | 0.682 | | 0.594 | | 0.635 | 0.709 | 0.647 | 0.677 | 0.525 | 0.599 | 0.560 | 0.408 | 0.821 | 0.545 | 0.467 | 0.872 | 0.609 | 0.642 | 0.554 | 0.595 |  |
|  | Overweight | 0.660 | | 0.624 | | 0.641 | 0.669 | 0.650 | 0.660 | 0.524 | 0.553 | 0.538 | 0.474 | 0.681 | 0.559 | 0.511 | 0.663 | 0.577 | 0.620 | 0.584 | 0.601 |  |
|  | Obese | 0.587 | | 0.600 | | 0.593 | 0.612 | 0.686 | 0.647 | 0.459 | 0.612 | 0.524 | 0.429 | 0.725 | 0.539 | 0.433 | 0.786 | 0.559 | 0.547 | 0.560 | 0.553 |  |
| Secondary One | Normal | 0.844 | | 0.895 | | 0.868 | 0.852 | 0.897 | 0.874 | 0.833 | 0.793 | 0.813 | 0.872 | 0.591 | 0.705 | 0.912 | 0.630 | 0.745 | 0.804 | 0.855 | 0.828 |  |
|  | Underweight | 0.624 | | 0.480 | | 0.542 | 0.645 | 0.515 | 0.573 | 0.448 | 0.520 | 0.481 | 0.346 | 0.737 | 0.470 | 0.384 | 0.854 | 0.530 | 0.584 | 0.440 | 0.502 |  |
|  | Overweight | 0.585 | | 0.531 | | 0.557 | 0.609 | 0.549 | 0.577 | 0.458 | 0.489 | 0.473 | 0.387 | 0.620 | 0.477 | 0.443 | 0.614 | 0.515 | 0.545 | 0.491 | 0.517 |  |
|  | Obese | 0.552 | | 0.463 | | 0.503 | 0.582 | 0.560 | 0.571 | 0.391 | 0.499 | 0.438 | 0.323 | 0.555 | 0.409 | 0.361 | 0.782 | 0.494 | 0.512 | 0.423 | 0.463 |  |
| Secondary Two | Normal | 0.835 | | 0.902 | | 0.867 | 0.843 | 0.906 | 0.873 | 0.831 | 0.780 | 0.805 | 0.870 | 0.565 | 0.685 | 0.909 | 0.581 | 0.709 | 0.795 | 0.862 | 0.827 |  |
|  | Underweight | 0.587 | | 0.397 | | 0.473 | 0.612 | 0.461 | 0.526 | 0.394 | 0.475 | 0.430 | 0.314 | 0.717 | 0.437 | 0.339 | 0.844 | 0.484 | 0.547 | 0.357 | 0.433 |  |
|  | Overweight | 0.516 | | 0.435 | | 0.472 | 0.540 | 0.422 | 0.474 | 0.378 | 0.423 | 0.399 | 0.327 | 0.589 | 0.420 | 0.365 | 0.579 | 0.448 | 0.476 | 0.395 | 0.432 |  |
|  | Obese | 0.474 | | 0.359 | | 0.409 | 0.497 | 0.464 | 0.480 | 0.283 | 0.404 | 0.333 | 0.240 | 0.491 | 0.323 | 0.284 | 0.748 | 0.412 | 0.434 | 0.319 | 0.369 |  |
| Secondary Three | Normal | 0.832 | | 0.906 | | 0.867 | 0.836 | 0.922 | 0.877 | 0.832 | 0.781 | 0.806 | 0.866 | 0.543 | 0.667 | 0.905 | 0.548 | 0.683 | 0.792 | 0.866 | 0.827 |  |
|  | Underweight | 0.579 | | 0.360 | | 0.444 | 0.612 | 0.425 | 0.501 | 0.393 | 0.463 | 0.425 | 0.304 | 0.686 | 0.421 | 0.330 | 0.823 | 0.471 | 0.539 | 0.320 | 0.404 |  |
|  | Overweight | 0.454 | | 0.382 | | 0.415 | 0.502 | 0.307 | 0.381 | 0.330 | 0.388 | 0.357 | 0.273 | 0.561 | 0.367 | 0.296 | 0.548 | 0.385 | 0.414 | 0.342 | 0.375 |  |
|  | Obese | 0.446 | | 0.317 | | 0.370 | 0.448 | 0.416 | 0.431 | 0.251 | 0.355 | 0.294 | 0.203 | 0.471 | 0.284 | 0.253 | 0.711 | 0.373 | 0.406 | 0.277 | 0.330 |  |
| Secondary Four | Normal | 0.820 | | 0.899 | | 0.858 | 0.820 | 0.920 | 0.867 | 0.811 | 0.757 | 0.783 | 0.846 | 0.527 | 0.649 | 0.896 | 0.516 | 0.655 | 0.780 | 0.859 | 0.818 |  |
|  | Underweight | 0.589 | | 0.377 | | 0.460 | 0.623 | 0.402 | 0.488 | 0.389 | 0.446 | 0.416 | 0.329 | 0.673 | 0.442 | 0.360 | 0.816 | 0.500 | 0.549 | 0.337 | 0.420 |  |
|  | Overweight | 0.410 | | 0.345 | | 0.375 | 0.437 | 0.247 | 0.315 | 0.282 | 0.354 | 0.314 | 0.232 | 0.518 | 0.321 | 0.249 | 0.534 | 0.340 | 0.370 | 0.305 | 0.335 |  |
|  | Obese | 0.358 | | 0.246 | | 0.291 | 0.371 | 0.329 | 0.349 | 0.188 | 0.275 | 0.223 | 0.172 | 0.438 | 0.247 | 0.223 | 0.708 | 0.339 | 0.318 | 0.206 | 0.251 |  |
| Secondary Five | Normal | 0.796 | | 0.887 | | 0.839 | 0.801 | 0.907 | 0.851 | 0.791 | 0.720 | 0.754 | 0.822 | 0.485 | 0.610 | 0.875 | 0.477 | 0.617 | 0.756 | 0.847 | 0.799 |  |
|  | Underweight | 0.573 | | 0.380 | | 0.457 | 0.611 | 0.442 | 0.513 | 0.399 | 0.478 | 0.435 | 0.352 | 0.686 | 0.465 | 0.386 | 0.809 | 0.522 | 0.533 | 0.340 | 0.417 |  |
|  | Overweight | 0.368 | | 0.281 | | 0.319 | 0.406 | 0.167 | 0.237 | 0.246 | 0.326 | 0.280 | 0.203 | 0.489 | 0.287 | 0.209 | 0.486 | 0.292 | 0.328 | 0.241 | 0.279 |  |
|  | Obese | 0.370 | | 0.224 | | 0.279 | 0.269 | 0.211 | 0.236 | 0.172 | 0.237 | 0.199 | 0.140 | 0.382 | 0.205 | 0.177 | 0.665 | 0.279 | 0.330 | 0.184 | 0.239 |  |
| Secondary Six | Normal | 0.770 | | 0.872 | | 0.818 | 0.777 | 0.891 | 0.830 | 0.753 | 0.690 | 0.720 | 0.798 | 0.478 | 0.598 | 0.855 | 0.483 | 0.617 | 0.730 | 0.832 | 0.778 |  |
|  | Underweight | 0.622 | | 0.444 | | 0.518 | 0.659 | 0.483 | 0.558 | 0.437 | 0.482 | 0.458 | 0.420 | 0.698 | 0.525 | 0.465 | 0.804 | 0.589 | 0.582 | 0.404 | 0.478 |  |
|  | Overweight | 0.341 | | 0.230 | | 0.275 | 0.355 | 0.151 | 0.212 | 0.222 | 0.328 | 0.265 | 0.184 | 0.479 | 0.266 | 0.186 | 0.449 | 0.263 | 0.301 | 0.190 | 0.235 |  |
|  | Obese | 0.146 | | 0.086 | | 0.108 | 0.333 | 0.243 | 0.281 | 0.133 | 0.186 | 0.155 | 0.109 | 0.329 | 0.164 | 0.115 | 0.571 | 0.192 | 0.106 | 0.046 | 0.068 |  |
|  |  | Primary Six Prediction Cohort | | | | | | | | | | | | | | | | | | | |  |
| Secondary One | Normal | 0.898 | | 0.917 | | 0.908 | 0.909 | 0.913 | 0.911 | 0.884 | 0.850 | 0.867 | 0.930 | 0.712 | 0.807 | 0.946 | 0.719 | 0.817 | 0.875 | 0.894 | 0.885 |  |
|  | Underweight | 0.715 | | 0.698 | | 0.706 | 0.719 | 0.708 | 0.713 | 0.598 | 0.622 | 0.610 | 0.522 | 0.758 | 0.619 | 0.543 | 0.738 | 0.626 | 0.692 | 0.675 | 0.683 |  |
|  | Overweight | 0.731 | | 0.667 | | 0.698 | 0.734 | 0.716 | 0.725 | 0.575 | 0.659 | 0.614 | 0.455 | 0.829 | 0.587 | 0.468 | 0.890 | 0.613 | 0.708 | 0.644 | 0.675 |  |
|  | Obese | 0.686 | | 0.627 | | 0.655 | 0.645 | 0.714 | 0.678 | 0.493 | 0.620 | 0.550 | 0.471 | 0.675 | 0.555 | 0.469 | 0.852 | 0.605 | 0.663 | 0.604 | 0.632 |  |
| Secondary Two | Normal | 0.877 | | 0.915 | | 0.896 | 0.887 | 0.912 | 0.900 | 0.865 | 0.829 | 0.847 | 0.911 | 0.666 | 0.770 | 0.938 | 0.668 | 0.780 | 0.855 | 0.893 | 0.874 |  |
|  | Underweight | 0.616 | | 0.572 | | 0.593 | 0.620 | 0.585 | 0.602 | 0.471 | 0.521 | 0.495 | 0.402 | 0.687 | 0.507 | 0.428 | 0.701 | 0.531 | 0.594 | 0.550 | 0.571 |  |
|  | Overweight | 0.675 | | 0.554 | | 0.609 | 0.689 | 0.606 | 0.645 | 0.494 | 0.566 | 0.528 | 0.392 | 0.767 | 0.518 | 0.408 | 0.851 | 0.552 | 0.653 | 0.532 | 0.587 |  |
|  | Obese | 0.602 | | 0.492 | | 0.541 | 0.579 | 0.569 | 0.574 | 0.408 | 0.479 | 0.441 | 0.380 | 0.592 | 0.463 | 0.386 | 0.769 | 0.514 | 0.580 | 0.470 | 0.519 |  |
| Secondary Three | Normal | 0.860 | | 0.910 | | 0.884 | 0.866 | 0.915 | 0.890 | 0.852 | 0.802 | 0.826 | 0.900 | 0.623 | 0.737 | 0.932 | 0.616 | 0.742 | 0.838 | 0.888 | 0.862 |  |
|  | Underweight | 0.533 | | 0.491 | | 0.511 | 0.574 | 0.481 | 0.523 | 0.385 | 0.458 | 0.419 | 0.330 | 0.632 | 0.433 | 0.348 | 0.648 | 0.453 | 0.511 | 0.469 | 0.489 |  |
|  | Overweight | 0.649 | | 0.480 | | 0.552 | 0.657 | 0.514 | 0.577 | 0.449 | 0.524 | 0.483 | 0.376 | 0.753 | 0.502 | 0.390 | 0.848 | 0.534 | 0.627 | 0.458 | 0.530 |  |
|  | Obese | 0.479 | | 0.396 | | 0.434 | 0.485 | 0.505 | 0.495 | 0.318 | 0.435 | 0.367 | 0.267 | 0.543 | 0.358 | 0.311 | 0.789 | 0.446 | 0.457 | 0.374 | 0.412 |  |
| Secondary Four | Normal | 0.837 | | 0.897 | | 0.866 | 0.846 | 0.905 | 0.875 | 0.833 | 0.783 | 0.807 | 0.875 | 0.587 | 0.703 | 0.918 | 0.587 | 0.716 | 0.815 | 0.875 | 0.844 |  |
|  | Underweight | 0.479 | | 0.431 | | 0.454 | 0.499 | 0.379 | 0.431 | 0.332 | 0.403 | 0.364 | 0.281 | 0.601 | 0.383 | 0.299 | 0.608 | 0.400 | 0.457 | 0.409 | 0.432 |  |
|  | Overweight | 0.599 | | 0.435 | | 0.504 | 0.634 | 0.495 | 0.556 | 0.439 | 0.500 | 0.467 | 0.374 | 0.718 | 0.492 | 0.402 | 0.834 | 0.542 | 0.577 | 0.413 | 0.482 |  |
|  | Obese | 0.456 | | 0.320 | | 0.376 | 0.451 | 0.465 | 0.458 | 0.264 | 0.386 | 0.313 | 0.244 | 0.500 | 0.328 | 0.287 | 0.768 | 0.418 | 0.434 | 0.298 | 0.354 |  |
| Secondary Five | Normal | 0.813 | | 0.890 | | 0.849 | 0.819 | 0.899 | 0.857 | 0.809 | 0.754 | 0.780 | 0.845 | 0.547 | 0.664 | 0.885 | 0.541 | 0.671 | 0.791 | 0.868 | 0.827 |  |
|  | Underweight | 0.420 | | 0.347 | | 0.380 | 0.434 | 0.249 | 0.317 | 0.291 | 0.380 | 0.330 | 0.232 | 0.547 | 0.326 | 0.249 | 0.561 | 0.345 | 0.398 | 0.325 | 0.358 |  |
|  | Overweight | 0.615 | | 0.442 | | 0.514 | 0.632 | 0.493 | 0.554 | 0.451 | 0.501 | 0.475 | 0.399 | 0.707 | 0.510 | 0.421 | 0.805 | 0.553 | 0.593 | 0.420 | 0.492 |  |
|  | Obese | 0.427 | | 0.264 | | 0.326 | 0.389 | 0.375 | 0.382 | 0.246 | 0.382 | 0.299 | 0.199 | 0.486 | 0.282 | 0.235 | 0.743 | 0.357 | 0.405 | 0.242 | 0.304 |  |
| Secondary Six | Normal | 0.786 | | 0.870 | | 0.826 | 0.801 | 0.878 | 0.838 | 0.776 | 0.729 | 0.751 | 0.820 | 0.534 | 0.647 | 0.866 | 0.547 | 0.671 | 0.764 | 0.848 | 0.804 |  |
|  | Underweight | 0.376 | | 0.329 | | 0.351 | 0.416 | 0.260 | 0.320 | 0.272 | 0.353 | 0.308 | 0.223 | 0.509 | 0.311 | 0.233 | 0.518 | 0.321 | 0.354 | 0.307 | 0.329 |  |
|  | Overweight | 0.634 | | 0.463 | | 0.535 | 0.650 | 0.537 | 0.588 | 0.477 | 0.519 | 0.497 | 0.441 | 0.720 | 0.547 | 0.478 | 0.790 | 0.596 | 0.612 | 0.441 | 0.513 |  |
|  | Obese | 0.250 | | 0.130 | | 0.171 | 0.261 | 0.174 | 0.209 | 0.174 | 0.232 | 0.199 | 0.142 | 0.377 | 0.206 | 0.173 | 0.681 | 0.277 | 0.228 | 0.108 | 0.149 |  |
| XG Boost eXtreme Gradient Boosting | | | | | | | | | | | | | | | | | | | | | |  |

***
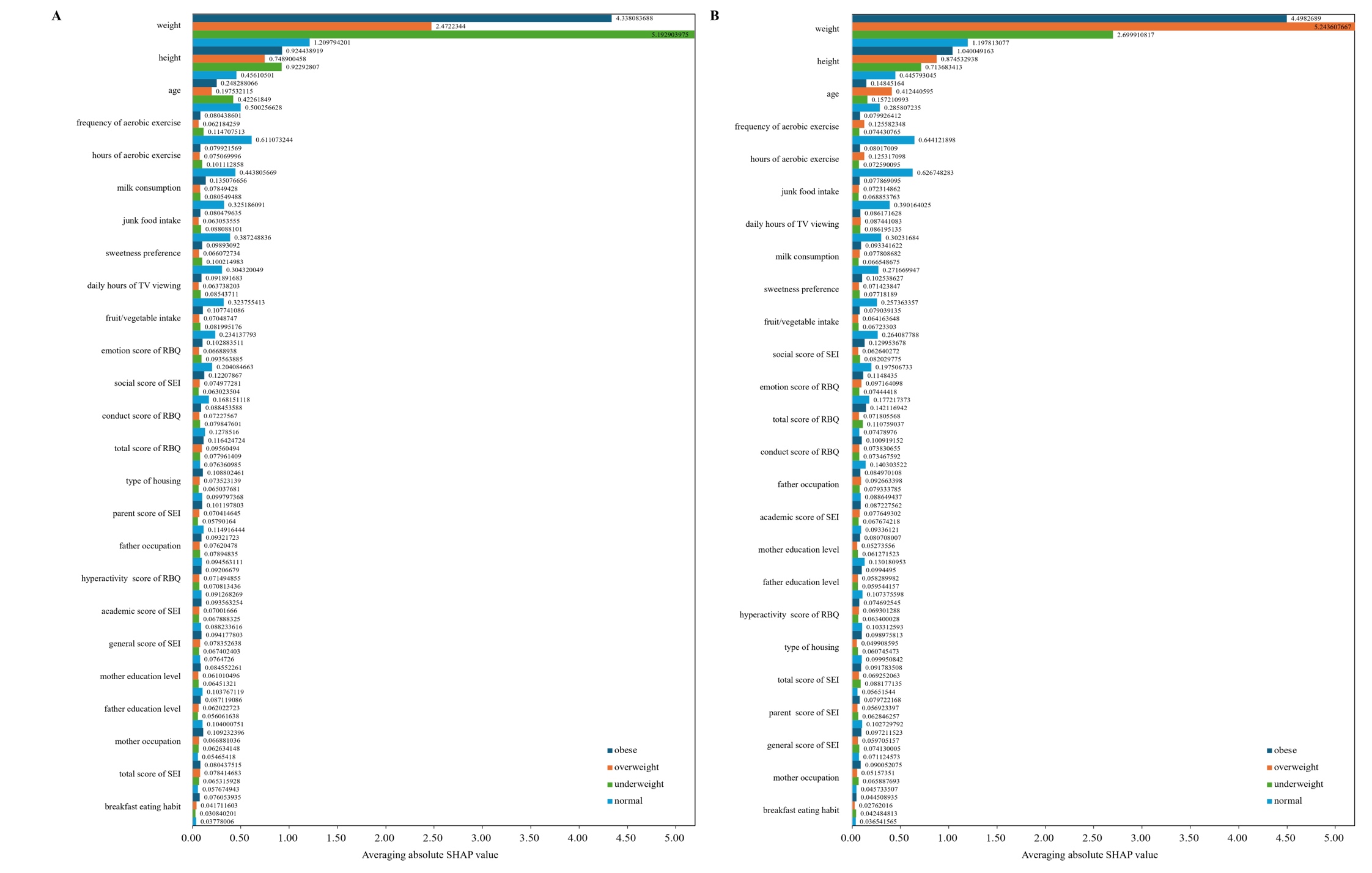
*Supple. Figure S1** Relative importance of predictors with weight status-specification for male group.

**A** based on the primary four cohort, **B** based on the primary six cohort. The relative predictor importance on each weight status was measured by the Shapley values under a XG Boost model. The predictors were ordered in descending order of overall importance.

*
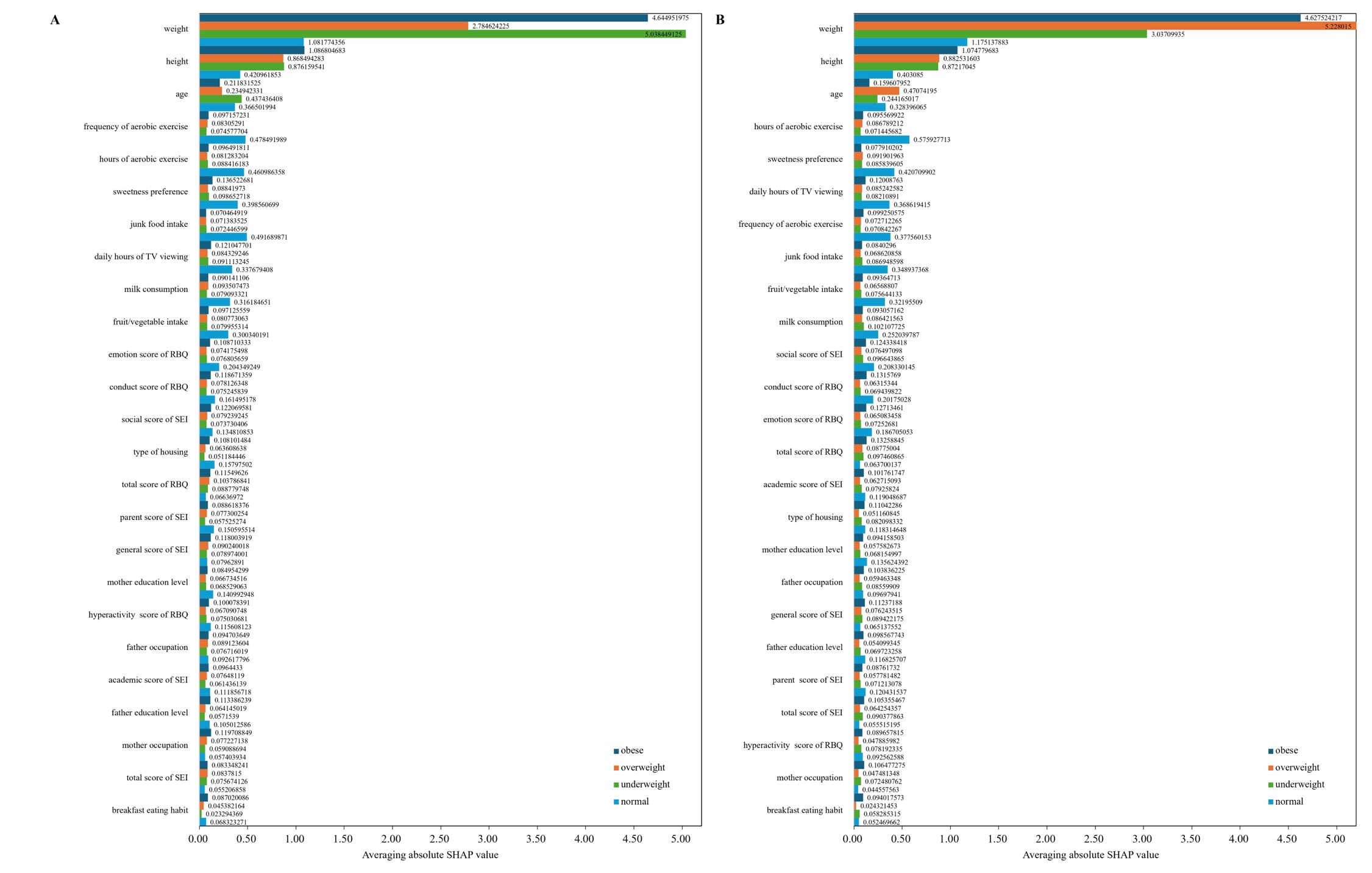
*

**Supple. Figure S2** Relative importance of predictors with weight status-specification for female group.

**A** based on the primary four cohort, **B** based on the primary six cohort. The relative predictor importance on each weight status was measured by the Shapley values under a XG Boost model. The predictors were ordered in descending order of overall importance.


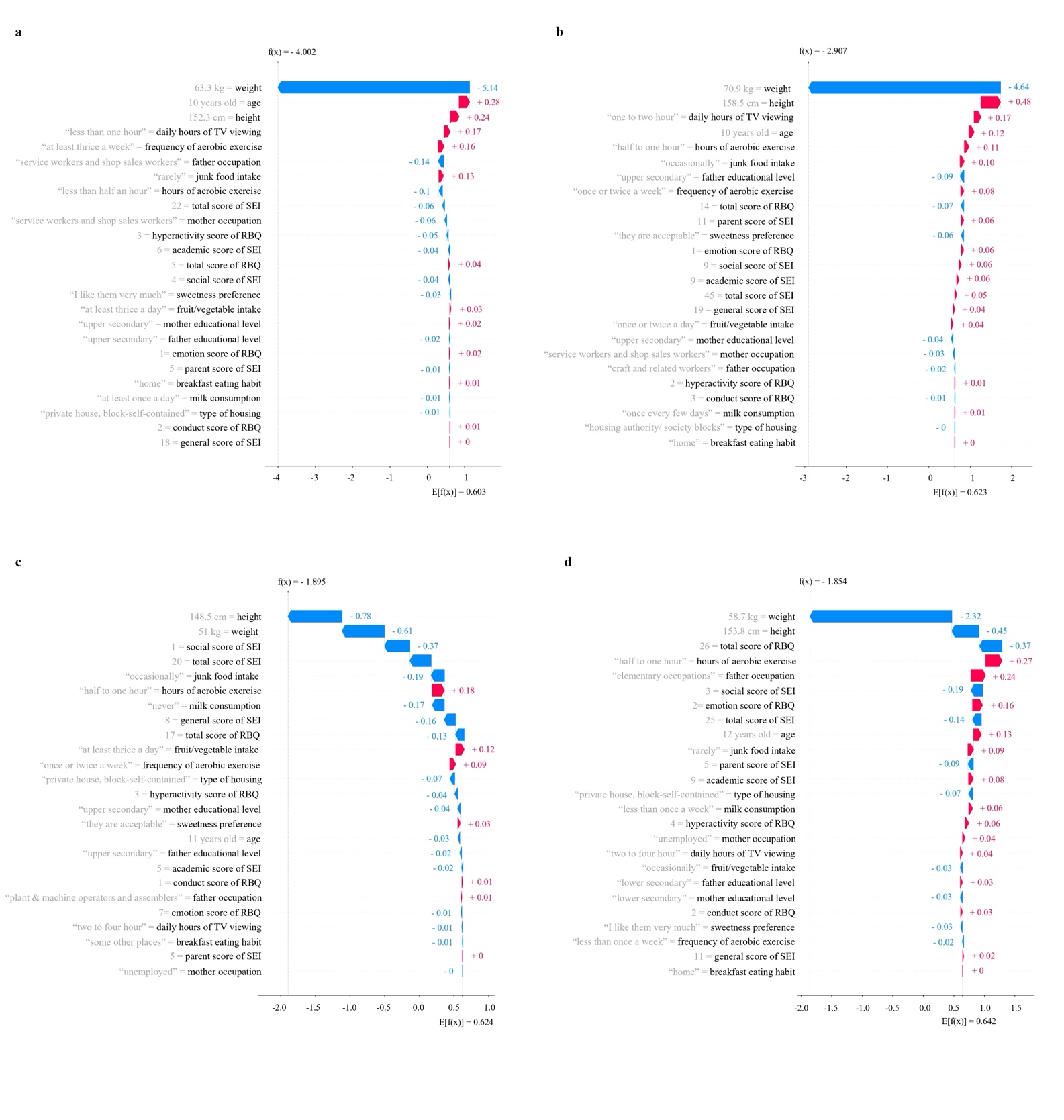


**Supple Figure S3** SHAP Waterfall plots of the predictors’ contribution to a correctly predicted weight status.

**a** Based on male child at primary four data, who is predicted to be obese at secondary two; **b** Based on a female child at primary four, who is predicted to be obese at secondary two; **c** Based on male child at primary six data, who is predicted to be obese at secondary four; **b** Based on a female child at primary six, who is predicted to be obese at secondary four. Each arrow shows the magnitude and direction a predictor’s contribution to the predicted outcome.
